# USP8 Promotes Smoothened Signaling by Preventing Its Ubiquitination and Changing Its Subcellular Localization

**DOI:** 10.1371/journal.pbio.1001238

**Published:** 2012-01-10

**Authors:** Ruohan Xia, Hongge Jia, Junkai Fan, Yajuan Liu, Jianhang Jia

**Affiliations:** Department of Molecular and Cellular Biochemistry, Markey Cancer Center, University of Kentucky, Lexington, Kentucky, United States of America; University of Zurich, Switzerland

## Abstract

The seven transmembrane protein Smoothened (Smo) is a critical component of the Hedgehog (Hh) signaling pathway and is regulated by phosphorylation, dimerization, and cell-surface accumulation upon Hh stimulation. However, it is not clear how Hh regulates Smo accumulation on the cell surface or how Hh regulates the intracellular trafficking of Smo. In addition, little is known about whether ubiquitination is involved in Smo regulation. In this study, we demonstrate that Smo is multi-monoubiquitinated and that Smo ubiquitination is inhibited by Hh and by phosphorylation. Using an in vivo RNAi screen, we identified ubiquitin-specific protease 8 (USP8) as a deubiquitinase that down-regulates Smo ubiquitination. Inactivation of USP8 increases Smo ubiquitination and attenuates Hh-induced Smo accumulation, leading to decreased Hh signaling activity. Moreover, overexpression of USP8 prevents Smo ubiquitination and elevates Smo accumulation, leading to increased Hh signaling activity. Mechanistically, we show that Hh promotes the interaction of USP8 with Smo aa625–753, which covers the three PKA and CK1 phosphorylation clusters. Finally, USP8 promotes the accumulation of Smo at the cell surface and prevents localization to the early endosomes, presumably by deubiquitinating Smo. Our studies identify USP8 as a positive regulator in Hh signaling by down-regulating Smo ubiquitination and thereby mediating Smo intracellular trafficking.

## Abstract 2

Hedgehog regulates the activity of its signal transducer Smoothened by enhancing its interaction with the deubiquitinase USP8, thereby promoting Smoothened translocation to the cell surface and so enhancing Hh signaling.

## Author Summary

The Hedgehog (Hh) signaling pathway is well known for its role in directing processes such as cell growth, proliferation, and differentiation during embryogenesis. The signal initiated by Hh binding to its receptor, Patched, is transduced by another protein called Smoothened (Smo), which moves from membranes inside the cell to accumulate on the cell surface when Hh binds. This accumulation of Smo on the cell surface is thought to play a central role in maintaining Hh signaling. In this study, we investigated how Hh controls the stability and movement of Smo inside the cell. We found that Smo is modified by addition of a small protein called ubiquitin (Ub), and that Hh regulates the ubiquitination of Smo. We identified an enzyme called USP8 that can remove the ubiquitin modification from Smo, thereby enhancing its signaling activity. Furthermore we show that Hh can enhance the interaction between Smo and USP8. Finally, we discovered that USP8 promotes the movement of Smo from inside the cell to the cell surface. We conclude that Hh promotes the deubiquitination of Smo by USP8, resulting in the relocation of Smo to the cell surface where it enhances Hh signaling.

## Introduction

Hedgehog (Hh) proteins function as morphogens and play critical roles in pattern formation and cell growth control. Hh signaling has also been implicated in tissue repair and stem cell maintenance [1]. Malfunction of Hh signaling causes birth defects as well as several types of cancer [2],[3]. The Hh signal is transduced through a receptor complex consisting of Patched (Ptc) and Ihog [4]. The seven transmembrane protein Smoothened (Smo) acts as a signal transducer, and the activity of Smo is inhibited by Ptc in the absence of Hh [5]–[7]. Binding of Hh to Ptc-Ihog relieves the inhibition of Smo by Ptc, which allows Smo to activate the cubitus interuptus (Ci)/Gli family of Zn-finger transcription factors and thereby induce the expression of Hh target genes, such as *decapentaplegic* (*dpp*), *ptc*, and *en* [5],[7]. Posttranslational regulation of the components in the Hh signaling pathway has been shown to be critical for their accumulation and activation. For example, phosphorylation of Smo and Ci/Gli has been extensively studied [8]. In *Drosophila*, it has been shown that the presence of Hh promotes the hyperphosphorylation and cell surface accumulation of Smo, whereas the absence of Hh allows for the hyperphosphorylation and proteolytic processing of Ci [5]. Although it has been shown that ubiquitination is important for Ci processing and degradation [9], it is not known whether ubiquitination is involved in the posttranslational regulation of other pathway components.

Hh induces the cell surface accumulation and phosphorylation of Smo [10] by multiple kinases, including protein kinase A (PKA) and casein kinase 1 (CK1) [11]–[13], which activate Smo by inducing a conformational change in the protein [14]. It has been shown that forced localization of Smo to the cell surface increases signaling activity, whereas endoplasmic retention of an activated form of Smo blocks activity [15]. The inhibition of endocytosis through the use of a dominant negative form of Rab5 promotes Smo cell surface accumulation [15]. In addition, an antibody uptake experiment using cultured *Drosophila* S2 cells suggested that Hh regulates Smo cell surface accumulation by blocking endocytosis and/or promoting the recycling of Smo [12]. Notably, Smo is primarily localized to the lysosomes of A-compartment cells in *Drosophila* imaginal discs, where Hh is not present, and is enriched on the plasma membrane of P-compartment cells where Hh stimulation occurs [16]. Smo has been established as a G protein-coupled receptor-like protein after the recent identification of an association with Gαi [17], as well as the finding that G protein-coupled receptor kinase 2 (Gprk2) phosphorylates and regulates Smo accumulation [18],[19]. Similar mechanisms have been proposed for the regulation of mammalian Smo, whereby both Smo and Ptc co-localize and internalize in endosomal compartments, and Hh induces the segregation of Smo away from Hh-Ptc complexes that are destined for lysosome degradation [20]. In addition, the association of Smo with β-arrestin 2 appears to promote Smo endocytosis through clathrin-coated pits [21]. Taken together, the controlled accumulation and localization of Smo in the Hh signaling pathway is thought to play a central role in maintaining signaling homeostasis. However, it is yet unclear how Hh controls the intracellular trafficking of Smo [3].

Ubiquitination is the enzymatic process by which proteins are covalently modified with the 76 amino acid protein ubiquitin (Ub). Ubiquitination has been shown to be important not only in the degradation of proteins, but also in the regulation of protein functions, such as protein activity, protein-protein interactions, and protein trafficking [22]–[24]. For example, cell surface receptor tyrosine kinases (RTKs) are monoubiquitinated at multiple sites, which mediates the internalization and subsequent transport of the proteins to the lysosome for destruction [25],[26]. The process of ubiquitination was shown to be a reversible modification after the identification of a large family of de-ubiquitinating enzymes (DUBs), which function by removing Ub conjugates from target proteins in order to regulate their biological activity and modulate intracellular trafficking [27]. It has recently been shown that deubiquitinating enzyme UBPY/ubiquitin-specific protease 8 (USP8) regulates the ubiquitination of Frizzled, which is the receptor for Wnt/Wingless [28]. In addition, deletion of UBPY/UBP8 in mice causes embryonic lethality [29]. However, in Hh signaling, it is currently not known whether the transmembrane protein Smo is regulated by ubiquitination and whether deubiquitination enzymes are involved in the sorting of Smo.

In this study, we found that Smo is monoubiquitinated, and this process is reduced by Hh or by phosphorylation. By using an RNAi screen that targets *Drosophila* DUBs in both the wings and cultured S2 cells, we identified USP8 as a DUB that prevents Smo ubiquitination and enhances the signaling activity of the protein. We also show that inactivation of USP8 by RNAi or by overexpressing a dominant negative form of USP8 increases Smo ubiquitination in S2 cells and prevents Smo accumulation in wing discs. The overexpression of USP8 down-regulated Smo ubiquitination and increased Smo accumulation. In addition, we found that USP8 is required for Hh-induced cell surface accumulation of Smo, and that Hh promotes the interaction between Smo and USP8 (see Figure S3). Moreover, we found that USP8 prevents Smo localization to early endosomes that are labeled with Rab5. Taken together, ubiquitination is likely a negative regulatory mechanism in Smo activation and USP8 plays an opposing role in this regulation.

## Results

### Smo Undergoes Monoubiquitination That Is Reduced by Hh or Phosphorylation

**Figure 1:**
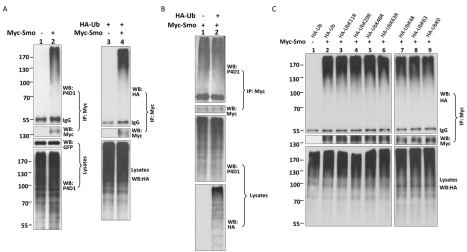
Multi-monoubiquitination of Smo in *Drosophila* S2 cells. (A) S2 cells were transfected with Myc-Smo alone or in combination with HA-Ub, followed by immunoprecipitation and Western blot analysis with the indicated antibodies. A blank vector was used as the immunoprecipitation control, GFP as the transfection control, and IgG as the loading control. (B) Myc-Smo was transfected in S2 cells with or without HA-Ub followed by immunoprecipitation and Western blot with the indicated antibodies. The expression of HA-Ub did not cause any changes in the levels of Smo ubiquitination. The levels of HA-Ub are comparable to the levels of free Ub detected by P4D1 (bottom panel). (C) Myc-Smo was co-transfected with HA-Ub or with each individual HA-tagged Ub mutant, followed by immunoprecipitation and Western blot analysis. HA-Ub and Ub mutants gave rise to a similar smear pattern (top panel).

In an attempt to explore whether Smo is regulated by ubiquitination, we examined Smo ubiquitination in S2 cells using an immunoprecipitation assay. We found that ubiquitinated Smo was readily detected by a commercial anti-Ub (P4D1) antibody, which detected the immunoprecipitated, endogenous Ub (Figure 1A, lane 2, top panel). Smo ubiquitination was also detected by an anti-hemagglutinin (HA) antibody in cells cotransfected with an HA-tagged Ub construct (HA-Ub) (Figure 1A, lane 4, top panel), and transfecting HA-Ub or HA-Ub mutants coincided with the pattern of the endogenous free Ub (Figure 1B, unpublished data). The smeared banding pattern of ubiquitinated Smo on SDS-PAGE suggested that Smo could be either polyubiquitinated or multi-monoubiquitinated. To differentiate these possibilities, we co-transfected S2 cells with a Myc-tagged Smo (Myc-Smo) together with various HA-Ub mutants and performed an immunoprecipitation assay to examine the pattern of Smo ubiquitination. As shown in Figure 1C, UbK0, a ubiquitin variant where all of the lysine (K) residues were changed to arginine (R), gave rise to an identically smeared banding pattern as wild-type Ub, K48-Ub, and K63-Ub (Figure 1C, lane 9, compare lanes 1, 7, and 8). This migration pattern was similar to the pattern of Ub variants containing individual lysine mutations (UbKR) (Figure 1C, lanes 3–6). These data suggest that Smo undergoes multi-monoubiquitination.

**Figure 2:**
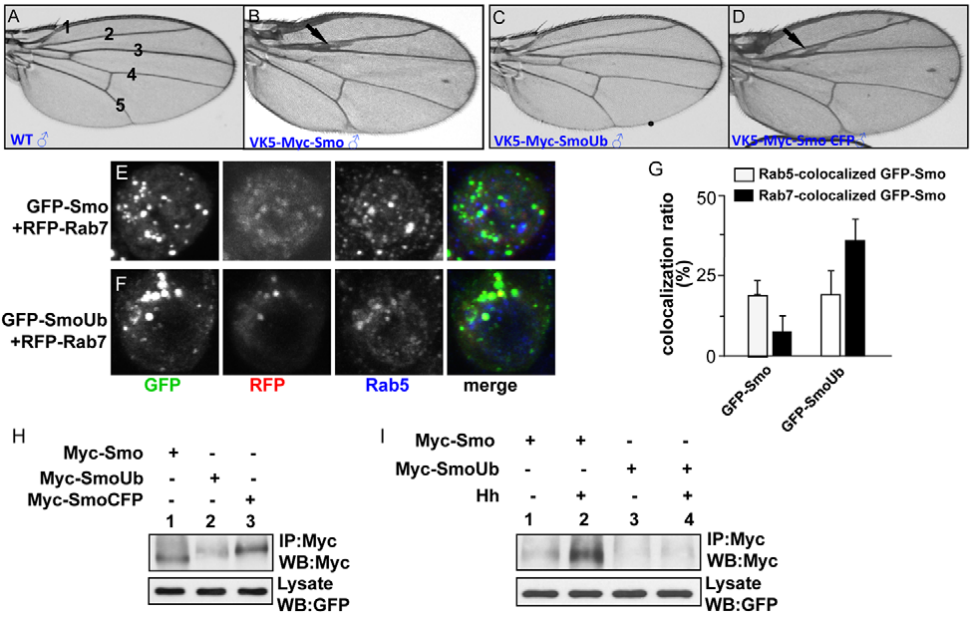
Addition of Ub to the C-terminus of Smo attenuates the signaling activity of Smo. (A) A wild-type adult wing indicates the five interveins. (B–D) Wings expressing Myc-Smo, Myc-SmoUb, or Myc-SmoCFP at the 75B1 attP locus by *MS1096* Gal4 to ensure equal expression of each individual constructs. The arrows in (B) and (D) indicate the extra wing structures in the anterior compartment of the wing, which is indicative of anterior ectopic Hh activity that is caused by the expression of wild-type Myc-Smo or Myc-SmoCFP. The wing in (C) shows the wild-type structure, which indicates that the activity of Myc-SmoUb is lower than that of Myc-Smo or Myc-SmoCFP. (E–F) S2 cells were transfected with RFP-Rab7 and GFP-Smo or GFP-SmoUb followed by immunostaining with Rab5 antibody to label the early endosomes. Late endosomes are labeled by RFP-Rab7. GFP indicates the expression and localization of Smo. The images shown in (E–F) are from intensity projection over the *z*-axis. (G) The percentage of Smo that co-localized with Rab5 or Rab7 compared to the total amount of Smo (GFP puncta) calculated from (E–F) (*n*≥10). More GFP-SmoUb puncta were localized in Rab7-labeled late endosomes compared to GFP-Smo. (H) S2 cells were transfected with Myc-Smo, Myc-SmoUb, or Myc-SmoCFP. Cell extracts were immunoprecipitated with the anti-Myc antibody and blotted with the anti-Myc antibody to examine the levels of Smo. GFP served as a transfection and loading control. Of note, the stability of Myc-SmoUb is much lower than that of Myc-Smo or Myc-SmoCFP. (I) S2 cells were transfected with Myc-Smo, Myc-SmoUb, or Myc-SmoCFP followed by treatment with or without Hh. Cell extracts were subjected to immunoprecipitation and Western blot with the anti-Myc antibody to detect the levels of Smo. Note that Hh stabilizes Myc-Smo (lane 2, top panel) but not Myc-SmoUb (lane 4, top panel). GFP served as a transfection and loading control.

We next wished to assess whether ubiquitination regulates Smo function in vivo. We constructed Myc-tagged Smo that had either a single Ub molecule or CFP cDNA fused in frame to the C-terminus of Smo (Myc-SmoUb and Myc-SmoCFP, respectively) and generated transgenic lines at the 75B1-VK5 attP locus to ensure equal expression of Myc-SmoUb, Myc-SmoCFP, and Myc-Smo [30]. Expression of Myc-Smo or Myc-SmoCFP induced an extra structure in Vein2 and Vein3, which was indicative of anterior ectopic Hh activity (Figure 2B and 2D). In contrast, expression of Myc-SmoUb resulted in a wild-type wing (Figure 2C). We further found that SmoUb preferentially localizes in the Rab7-labeled late endosomes (Figure 2E–G), and that SmoUb was less stable (Figure 2H) and had less responsiveness to Hh stimulation (Figure 2I). These data suggest that ubiquitination plays a negative role in Smo regulation.

**Figure 3:**
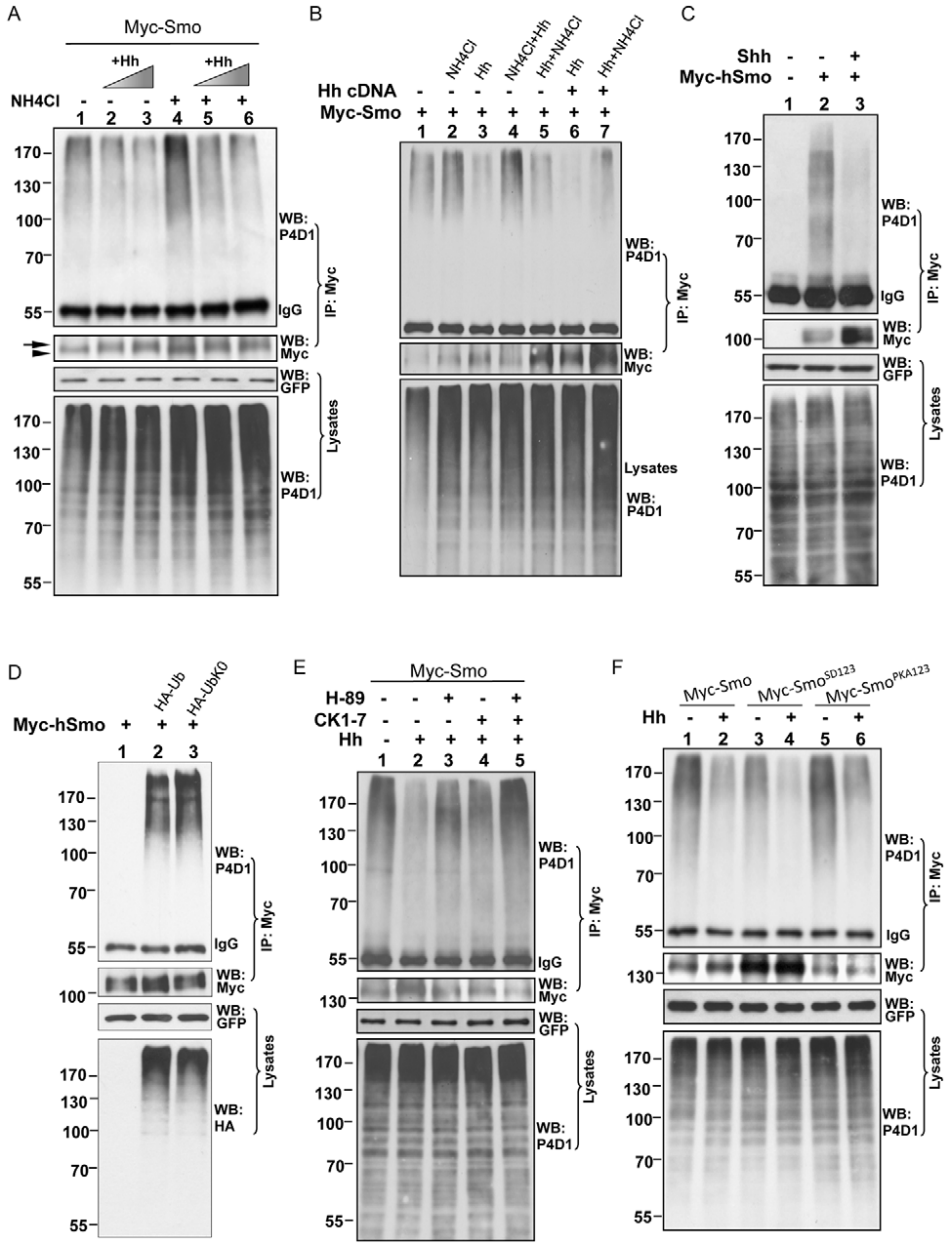
Smo ubiquitination is regulated by Hh and phosphorylation. (A) S2 cells were transfected with Myc-Smo and treated with 30% (lanes 2 and 5) or 60% (lanes 3 and 6) of Hh-conditioned medium or control medium (lanes 1 and 4), followed by treatment with or without NH_4_Cl. Cell extracts were immunoprecipitated and a Western blot was performed with the indicated antibodies. IgG served as a loading control and GFP was used as the transfection control. The arrow indicates hyperphosphorylated forms of Smo and the arrowhead indicates hypophosphorylated and unphosphorylated forms. (B) Sequential treatments with Hh and lysosomal inhibitor induce differential ubiquitination of Smo. S2 cells were transfected with Myc-Smo and treated with either Hh or the lysosomal inhibitor NH4Cl alone, or in combinations of Hh and NH4Cl treatment, followed by immunoprecipitation and Western blot with the indicated antibodies to examine the ubiquitination levels of Smo. The following treatments were performed: lane 1, control; lane 2, treatment with NH4Cl at a final concentration of 10 mM for 24 h; lane 3, treatment with 60% HhN-conditioned medium for 24 h; lane 4, treatment with NH4Cl at the concentration of 10 mM for the first 6 h, then adding 60% of the volume of HhN-conditioned medium, keeping NH4Cl at a final concentration of 10 mM for 18 h; lane 5, treatment with 60% volume of HhN-conditioned medium for the first 6 h, then adding NH4Cl at final concentration of 10 mM, keeping 60% volume of Hh medium for 18 h; lanes 6 and 7, HhN cDNA was cotransfected to achieve higher levels of Hh treatment. Consistently, Hh treatment down-regulates (lane 3, top panel) whereas NH4Cl treatment up-regulates (lane 2, top panel) Smo ubiquitination. However, Hh treatment down-regulates Smo ubiquitination before NH4Cl treatment (lane 5, top panel), but not after NH4Cl treatment (lane 4, top panel). The further down-regulation of Smo ubiquitination by Hh was achieved by cotransfecting Hh cDNA (lane 6, compared to lane 3). (C) NIH3T3 cells were transfected with Myc-hSmo and treated with or without recombinant Shh. Shh induced a marked decrease in hSmo ubiquitination (lane 3). (D) HEK293T cells were transfected with the indicated constructs and cell extracts were immunoprecipitated and blotted with the indicated antibodies. Cell lysates were also subjected to direct Western blot to examine the HA-tagged Ub expression. GFP served as transfection and loading control. Human Smo had a similar multi-mono ubiquitination pattern compared to fly Smo. (E) S2 cells transfected with Myc-Smo were treated with Hh-conditioned medium or control medium in combination with a PKA inhibitor (H-89) or CK1 inhibitor (CK1-7). Immunoprecipitation and Western blots were performed with the indicated antibodies. The levels of ubiquitinated Smo were increased by inactivating kinases. (F) S2 cells were transfected with wild-type, phospho-mimetic, or phospho-deficient Smo followed by treatment with Hh-conditioned medium or control medium. The same ubiquitination assay was carried out to examine ubiquitinated Smo with IgG and GFP serving as controls.

To explore whether Hh regulates Smo ubiquitination, we treated cells with varying concentrations of Hh-conditioned medium and analyzed the results in the ubiquitination assay. An increase in the concentration of Hh resulted in a reduction of ubiquitinated Smo (Figure 3A, compare lanes 2–3 to lane 1, top panel), indicating that Hh down-regulated Smo ubiquitination. We also found that the treatment of cells with ammonium chloride (NH_4_Cl), a lysosomal inhibitor, increased both the ubiquitination (Figure 3A, compare lane 4 to lane 1, top panel) and total protein (Figure 3A, compare lane 4 to lane 1, upper-middle panel) levels of Smo, suggesting that the lysosome may mediate the degradation of ubiquitinated Smo. These results are consistent with the finding that Smo accumulates in the lysosome in the absence of Hh [16]. Treatment with Hh followed by NH_4_Cl dramatically reduced Smo ubiquitination (Figure 3A, lanes 5–6, top panel; Figure 3B, lanes 5 and 7, top panel), whereas treatment with NH_4_Cl followed by Hh had very little effect on Smo ubiquitination (Figure 3B, lane 4, top panel). These data suggest that Hh likely down-regulates Smo ubiquitination before Smo enters the lysosome. The Hh-induced mobility shifts and stabilization of Myc-Smo (Figure 2A, upper-middle panel; Figure 2B, middle panel) are consistent with our previous findings [12],[30],[31].

To determine if mammalian Smo is regulated by ubiquitination, we transfected NIH3T3 cells with Myc-tagged human Smo (Myc-hSmo) and performed a similar immunoprecipitation assay as used for *Drosophila* S2 cells. We found that hSmo underwent ubiquitination, which was detected by the P4D1 antibody (Figure 3C, lane 2, top panel). In addition, Shh treatment induced a substantial decrease in hSmo ubiquitination (Figure 3C, lane 3, top panel). To examine whether hSmo is also multi-monoubiquitinated, we performed an immunoprecipitation assay in HEK293T cells transfected with Myc-hSmo and HA-Ub constructs. We found that Myc-hSmo pulled bound both HA-Ub and HA-UbK0 in a similar pattern (Figure 3D, top panel). These data suggest a conserved mechanism that regulates mammalian Smo.

It has previously been shown that Hh-induced phosphorylation by PKA and CK1 promotes *Drosophila* Smo cell-surface accumulation and signaling activity [11]–[13]. Our data suggested that Hh prevents Smo ubiquitination and that ubiquitination may down-regulate Smo activity, so we therefore investigated whether there was any relationship between Smo phosphorylation and ubiquitination. As shown in Figure 2E, the Hh-induced reduction of Smo ubiquitination was compromised by treatment with H-89 or CK1-7, which are inhibitors of PKA and CK1, respectively (Figure 3E, compare lanes 3, 4, or 5 to lane 2, top panel). Consistently, mutating the main isoform of PKAc in wing disc attenuated Smo accumulation and elevated the levels of protein ubiquitination (Figure S1) [12]. We further found that a Smo phospho-mimetic mutation (Smo^SD123^) that had mutations in the PKA and CK1 phosphorylation sites [12] had higher levels of Smo protein but markedly lower levels of Smo ubiquitination (Figure 3F, compare lane 3 to lane 1, top panel), whereas the phosphorylation-deficient form of Smo (Smo^PKA123^) had lower levels of Smo protein but higher levels of Smo ubiquitination (Figure 3F, compare lane 5 to lane 1, top panel). Moreover, Hh down-regulated the ubiquitination of Smo variants (Figure 3F, top panel), suggesting that phosphorylation at other sites in the Smo C-tail [13] could counteract further ubiquitination of Smo. Taken together, our observations suggest that phosphorylation antagonizes ubiquitination in the regulation of Smo.

### Identification of a Deubiquitinase for Smo by an RNAi Screen

**Figure 4:**
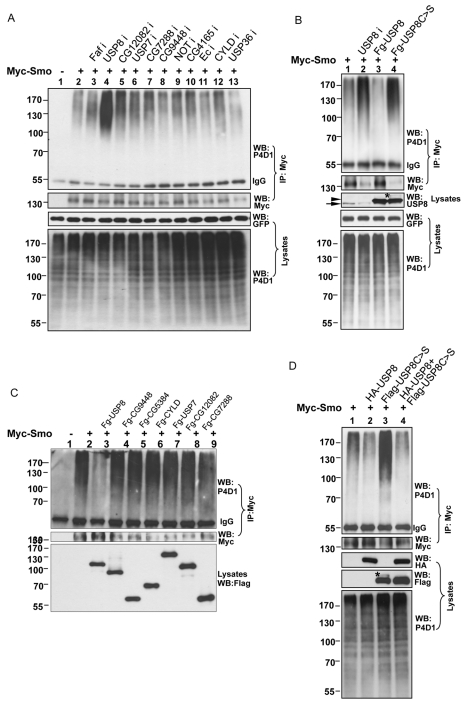
Identification of USP8 as a DUB for Smo. (A) S2 cells were transfected with Myc-Smo and treated with the indicated DUB dsRNA, followed by immunoprecipitation with an anti-Myc antibody and Western blot with the P4D1 antibody to examine the levels of Smo ubiquitination. (B) Myc-Smo was transfected either alone or in combination with Flag-USP8, Flag-USP8C>S, or USP8 dsRNA treatment. The immunoprecipitation assay was performed to examine the amount of ubiquitinated Smo. The efficiency of USP8 RNAi was examined with the anti-USP8 antibody. The arrowhead indicates transfected USP8 and the arrow indicates endogenous USP8. Notably, USP8 RNAi and USP8C>S severely destabilized Smo, whereas USP8 stabilized Smo in S2 cells. (C) S2 cells were transfected with Myc-Smo and the indicated DUB constructs followed by immunoprecipitation with the anti-Myc antibody and Western blot with the anti-Ub P4D1 antibody. Flag-USP8, but not other DUBs, reduced Smo ubiquitination (lane 3, compared to lane 2, top panel). Cell lysates were also subjected to Western blot to examine the expression of different DUBs (bottom panel). (D) S2 cells were transfected with the indicated constructs and an immunoprecipitation assay was performed to detect Smo ubiquitination. The asterisks in (B) and (D) show a possible modification on USP8C>S, which was down-regulated by the co-transfection of USP8, suggesting the possibility of ubiquitination.

Ubiquitin ligases and DUBs play opposing roles in the regulation of protein ubiquitination. To explore whether DUBs were involved in the regulation of Smo, we obtained 45 RNAi lines from the Vienna *Drosophila* RNAi Center (VDRC), which target 33 potential DUBs in the *Drosophila* genome (Table S1) [32],[33], and performed an in vivo screen by overexpressing individual RNAi lines through the wing-specific *MS1096* Gal4 to assess for the induction of an adult wing phenotype. The screen showed that RNAi-mediated knockdown of 11 genes affected wing development, as indicated by wing phenotypes or wing blisters (Table S1; unpublished data). Since it was possible that a specific DUB may reduce Smo ubiquitination and cause changes in Smo accumulation, we immunostained the wing imaginal discs using an anti-SmoN antibody when the expression of each of the 11 DUB genes was knocked down by RNAi. Expression of *UAS-RNAi* lines that targeted USP8 caused a reduction in Hh-induced Smo accumulation in P-compartment cells. Moreover, the expression of Hh target genes, such as *ptc*, was attenuated in the dorsal compartment cells where the *ap*-Gal4 was expressed. We also examined the levels of Smo ubiquitination in S2 cells when the expression of each of the 11 DUBs was knocked down by RNAi. As shown in Figure 4A, inactivation of USP8 in S2 cells by RNAi dramatically enhanced the levels of Smo ubiquitination (Figure 4A, lane 4, compare to lane 2, top panel), whereas the reduction of other DUBs by RNAi did not, suggesting that the inhibition of Smo accumulation by USP8 RNAi in wing discs was due to the increase in Smo ubiquitination. We then overexpressed Myc-Smo with each of the DUBs in S2 cells and performed an immunoprecipitation assay to examine the levels of Myc-Smo ubiquitination. As shown in Figure 4C, USP8, but not the other DUBs, reduced the ubiquitination of Myc-Smo. Together, these data suggest that USP8 is a specific DUB for Smo.

DUBs have a highly conserved motif (GNTCYMNS) in the catalytic domain, and mutation of the catalytic Cys in this sequence often leads to an inactive form of the enzyme [33]. To further examine the role of USP8 in Smo regulation, we generated a Flag-tagged USP8 mutant with Cys572 changed to Ser (Flag-USP8C>S) and co-transfected Myc-Smo with Flag-USP8C>S, Flag-USP8, or USP8 dsRNA in S2 cells. Overexpression of Flag-USP8 in S2 cells reduced Smo ubiquitination (Figure 4B, lane 3, top panel), whereas knockdown of USP8 by RNAi enhanced the levels of ubiquitinated Smo (Figure 4B, lane 2, top panel), which was consistent with the data from the screen. We also found that the overexpression of Flag-USP8C>S did not reduce Smo ubiquitination (Figure 4B, lane 4, top panel), suggesting that USP8C>S was an inactive enzyme. In contrast, USP8C>S increased Smo ubiquitination, which was similar to the phenotype induced by the RNAi-mediated knockdown of USP8 (Figure 4B, lanes 2 and 4, compare to lane 1, top panel). These results suggested that USP8C>S may have a dominant negative effect on endogenous USP8. In agreement with this hypothesis, the overexpression of HA-USP8 reversed the effects of Flag-USP8C>S and again reduced the levels of ubiquitinated Smo (Figure 4D, lane 4, top panel). These results were consistent with the previous finding that a consensus Cys mutation in USP8 caused dominant negative effects in cultured vertebrate cells [34].

### USP8 Regulates Smo Ubiquitination

**Figure 5:**
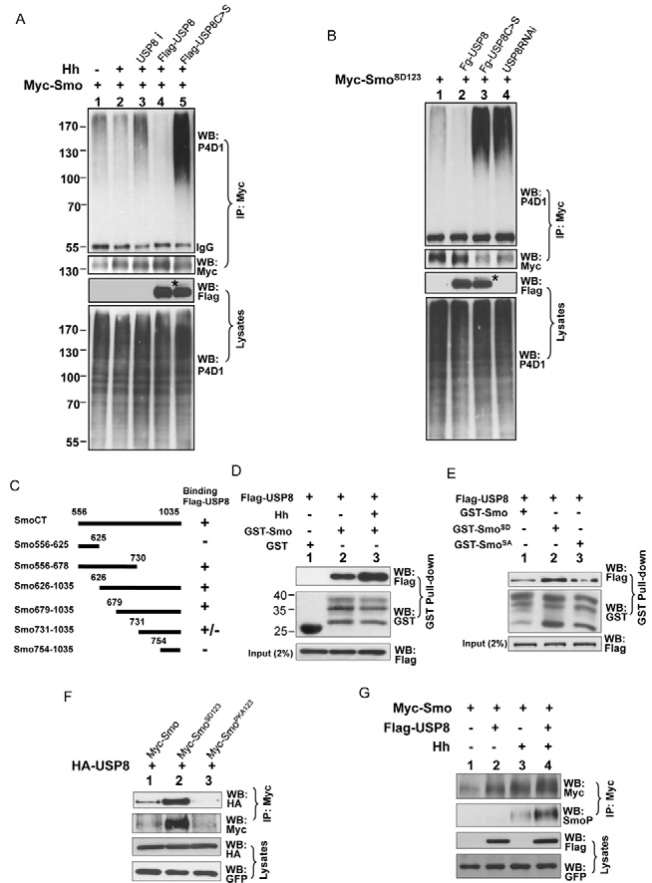
USP8 regulates Smo ubiquitination. (A) S2 cells transfected with Myc-Smo in combination with the indicated USP8 constructs or with USP8 dsRNA treatment were treated with Hh-conditioned medium or control medium. An immunoprecipitation assay was carried out with the indicated antibodies to examine the regulation of Smo ubiquitination by Hh or USP8. In the above experiments, GFP served as a transfection and loading control. (B) S2 cells were transfected with Myc-Smo^SD123^ alone or in combination with either Flag-USP8, Flag-USP8C>S, or USP8 dsRNA treatment. Cell extracts were immunoprecipitated with the anti-Myc antibody and subjected to a Western blot with the anti-Ub P4D1 antibody to detect ubiquitinated Smo (top panel), or with the anti-Myc antibody to detect the expression of Myc-Smo^SD123^ (upper-middle panel). Cell lysates were also subjected to a direct Western blot with the anti-Flag antibody to detect USP8 or USP8C>S overexpression (lower-middle panel) or with the anti-Ub P4D1 antibody to detect the overall level of Ub (bottom panel). The asterisk indicates a possible modification of USP8C>S. USP8 reduced Smo^SD123^ ubiquitination, whereas USP8C>S enhanced Smo^SD123^ ubiquitination. The asterisks in (A) and (B) show a possible modification on USP8C>S. (C) The interaction between Smo fragments and USP8 was determined in S2 cells. Briefly, S2 cells were co-transfected with Flag-USP8 and each Myc-Smo construct followed by immunoprecipitation with the anti-Myc antibody and Western blot with anti-Flag antibody to detect the Smo-bound USP8. Smo1-555 (N-terminal part of Smo) did not interact with USP8 (not shown). The +/− indicates a weak interaction. (D) Hh up-regulates Smo-USP8 interaction. Extracts from S2 cells expressing Flag-USP8 with or without Hh treatment were incubated with the bacterially expressed GST or GST-Smo625-753 fusion proteins. The bound USP8 proteins were analyzed by Western blot with the anti-Flag antibody. The middle panel shows the GST and GST-Smo fusion proteins. The lower panel indicates the equal amount of input USP8. (E) The interaction between Flag-USP8 and GST-Smo625-753, GST-Smo625-753^SD123^ (GST-Smo^SD^, with PKA and CKI1 sites mutated to Asp), and GST-Smo625-753^PKA123^ (GST-Smo^SA^, with PKA sites mutated to Ala). (F) S2 cells were co-transfected with HA-USP8 and wild-type, phospho-mimetic, or phospho-deficient Smo, followed by immunoprecipitation and Western blot analysis to examine the interaction between Smo and USP8. GFP served as a transfection and loading control. (G) S2 cells were transfected with Myc-Smo alone or in combination with Flag-USP8 and treated with Hh-conditioned medium or control medium. Cell extracts were immunoprecipitated with the anti-Myc antibody and blotted with either anti-Myc to detect Smo phosphorylation, indicated by its mobility shift on the SDS gel, or anti-SmoP, to directly detect Smo phosphorylation. The overexpression of USP8 increased the level of Smo without any effect on Smo phosphorylation. GFP served as a transfection and loading control.

To determine whether USP8 has a role in the Hh-mediated reduction of Smo ubiquitination, we examined Smo ubiquitination when USP8 was inactivated. As shown in Figure 5A, Smo ubiquitination was consistently reduced by Hh treatment (Figure 5A, lane 2, top panel). However, the reduction of Smo ubiquitination was not observed when USP8 was knocked down by RNAi or inactivated by expressing USP8C>S (Figure 5A, lanes 3 and 5, top panel). In contrast, overexpression of Flag-USP8 caused a greater reduction in Smo ubiquitination (Figure 5A, lane 4, top panel). These data suggest that USP8 is required for the Hh-induced reduction of Smo ubiquitination. We also wondered whether USP8 regulates the ubiquitination of Smo^SD123^. We found that when Smo^SD123^ was co-expressed with Flag-USP8, the level of Smo ubiquitination was reduced, whereas when Smo^SD123^ was co-expressed with USP8RNAi or Flag-USP8C>S, the levels of Smo ubiquitination were increased (Figure 5B). These results suggested that the ubiquitination of Smo^SD123^ can be regulated by USP8.

To test whether USP8 regulates Smo ubiquitination by interacting with Smo, we carried out co-immunoprecipitation experiments and found that Myc-SmoCT (Smo C-tail), but not Myc-SmoCT (Smo lacking its C-tail), co-immunoprecipitated with HA-USP8 (Figure S2B). To further map the Smo domain responsible for interacting with USP8, we tested various SmoCT truncations, which were previously generated (Figure 5C) [30], for their ability to bind USP8. We found that amino acids 625–753 of Smo were responsible for the interaction with USP8 (Figure 5C, unpublished data).

Hh might prevent Smo ubiquitination by regulating the DUB. However, it is unlikely that USP8 activity per se is regulated by Hh, as USP8 has been shown to be involved in multiple signaling pathways. The finding that USP8 interacted with Smo led to the hypothesis that Hh might control the accessibility of Smo to the DUB. To test this, we used a GST pull-down assay previously described [30]. We found that GST-Smo625-753 pulled down more Flag-USP8 in the presence of Hh (Figure 5D, lane 3, top panel) than that in the absence of Hh, suggesting that Hh regulates the accessibility of USP8 to Smo. We also found that phosphor-mimetic mutation in GST-Smo enhanced its interaction with USP8 (Figure 5E, lane 2, top panel). In addition, we found, in an immunoprecipitation experiment, that a substantial amount of Smo^SD123^ interacted with USP8 (Figure 5F, lane 2, top panel). In contrast, Smo^PKA123^ barely interacted with USP8 (Figure 5F, lane 3, top panel). These data suggest that phosphorylation of Smo promotes the formation of a Smo-USP8 complex, which may amplify the Hh stimulation and lead to the activation of Smo.

To better address the relationship between phosphorylation and deubiquitination, we used a newly developed phospho-Smo antibody to specifically detect Smo phosphorylation at the 2^nd^ cluster (anti-SmoP, Fan et al., unpublished). We found that USP8 stabilized Smo (Figure 5G, lane 2, compared to lane 1, top panel), but neither promoted Smo mobility shift on the gel (Figure 5G, lane 2, top panel) nor induced Smo phosphorylation (Figure 5G, lane 2, upper middle panel). Consistently, Hh induced Smo phosphorylation, which was detected by the mobility shift and the anti-SmoP antibody (Figure 5G, lane 3, top and upper middle panels). Interestingly, in the presence of Hh and USP8, Smo gave rise to a peak level of phosphorylation (Figure 5G, lane 4, top and upper middle panels). These observations suggest that USP8 does not primarily regulate Smo phosphorylation but promotes Smo phosphorylation when Hh is present.

### USP8 Promotes Smo Accumulation in Wing Imaginal Discs

**Figure 6:**
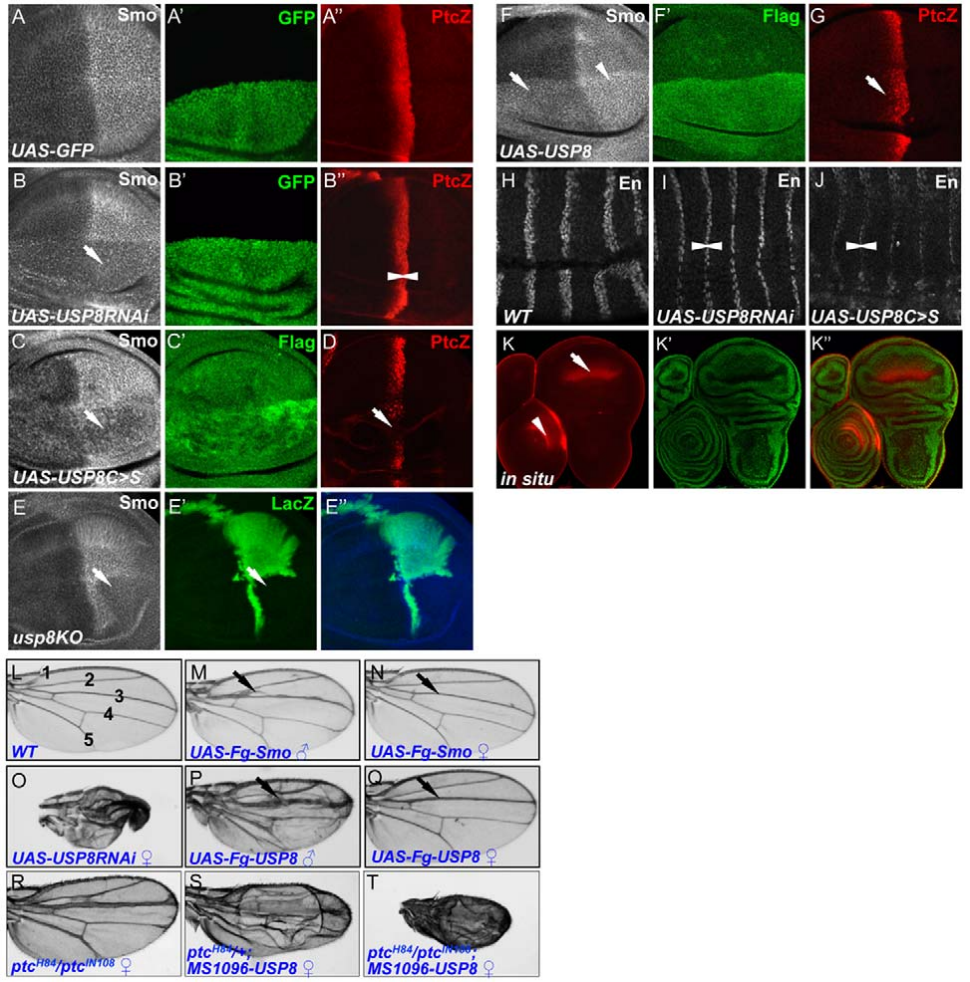
The function of USP8 in regulating Smo accumulation in the *Drosophila* wing. (A–A″) A wing disc expressing *UAS-GFP* by the dorsal compartment-specific *ap*-Gal4 was stained for Smo and ptc-lacZ to show the wild-type staining. (B–B″) A wing disc from flies co-expressing *UAS-USP8RNAi* and *UAS-GFP* by *ap*-Gal4 was stained for Smo and ptc-lacZ. The arrow in (B) indicates the attenuated accumulation of Smo and the arrowheads indicate the down-regulation of ptc-lacZ. GFP indicates that *ap*-Gal4 is expressed in the dorsal compartment cells of the wing disc. (C–D) Wing discs expressing *UAS-Flag-USP8C>S* by *MS1096* Gal4 were stained for Smo (grey in C), Flag (green in C′), and ptc-lacZ (red in D). The arrow in (C) indicates inhibition of Smo accumulation. The arrow in (D) indicates attenuated *ptc-lacZ* expression. Note that the level of Gal4 driven by MS1096 is higher in the dorsal compartment than in the ventral compartment. (E–E″) A wing disc bearing *usp8KO* homozygous clones, which were marked by the lack of β-gal staining, was immunostained for Smo. The arrow in (E) shows the reduced Smo accumulation in P-compartment cells. (F–G) Wing discs expressing *UAS-Flag-USP8* by *MS1096* Gal4 were stained for Smo (grey in F), Flag (green in F′), and ptc-lacZ (red in G). Arrow and arrowhead in (F) indicate the elevated Smo and arrow in (G) indicates the expansion of ptc-lacZ. (H) A wild-type embryo was stained for En. (I–J) Embryos expressing USP8RNAi or USP8C>S by *act5C*-Gal4 were immunostained for En. The arrowheads indicate the reduction and contraction of En expression. (K–K″) USP8 expression patterns in wing and leg discs (arrow and arrowhead, respectively) were determined by FISH with a DIG-labeled mRNA probe against USP8. Rhodamin (red) was used to detect the DIG-labeled mRNA, and DAPI (green, was false colored in green to provide better contrast) was used to label the nuclei. All wing imaginal discs shown in this study were oriented with anterior on the left and ventral on the top. (L) A wild-type adult wing showing interveins 1–5. (M–N) Wings from either male or female flies expressing Flag-Smo by *MS1096* Gal4. The arrow in (M) indicates the overgrowth structures between Vein 2 and 3, and the arrow in (N) indicates the thickened Vein 3 caused by the overexpression of Smo. (O) A wing from flies expressing USP8RNAi by *MS1096* Gal4. (P–Q) Wings from male or female flies expressing Flag-USP8 by *MS1096* Gal4. Arrows indicate the wing overgrowth structure or the thickness of Vein 3. Of note, males of the same genotype have more severe phenotypes than females, presumably due to dosage compensation of the X-chromosome carrying the *MS1096*-Gal4. (R) A wing from *ptc* mutant flies indicates the overgrowth between Vein 2 and 3. (S–T) Adult wings from flies expressing Flag-USP8 by *MS1096* Gal4 in the background of *ptc* mutants. In the *ptc* heterozygous or homozygous background, USP8 induced more severe wing overgrowth, which is indicated by wing blisters (S) or sick wings (T).

We next performed both loss- and gain-of-function studies in wing discs to examine the regulation of Smo accumulation by USP8. We found that the dominant negative USP8C>S blocked Smo accumulation in P-compartment cells (Figure 6C) and attenuated *ptc-lacZ* expression (Figure 6D), which is a phenotype similar to that caused by RNAi of USP8 (Figure 6B–B″). To further examine the physiological function of USP8 in regulating Smo, we also used an *usp8* hypomorphic allele, *usp8KO*, that has been previously described in the literature [28]. Smo accumulation was severely reduced in *usp8KO* cells (Figure 6E), and pupa bearing *usp8KO* clones were lethal (unpublished data). These loss-of-function studies indicate that USP8 is required for Smo accumulation. In addition, a gain-of-function experiment showed that the overexpression of USP8 caused an accumulation of Smo in both A- and P-compartment cells (Figure 6F) and caused anterior expansion of *ptc-lacZ* in cells that received Hh (Figure 6G). Moreover, inactivation of USP8 by either USP8RNAi or USP8C>S in *Drosophila* embryo down-regulated En expression (Figure 6I–J, compare to wild-type En staining in H) and caused lethality of the embryo or pupa (unpublished data). We also examined the expression pattern of endogenous USP8 transcription by in situ hybridization and found that USP8 was highly expressed in the wing pouch that is more sensitive to Hh signaling (Figure 6K–K″). Our observations strongly suggested that USP8 has a positive role in Hh-induced Smo accumulation, which is likely due to the role in regulating Smo ubiquitination.

Our previous study showed that overexpression of *UAS-Smo* by *MS1096* Gal4 caused albeit low levels of ectopic Hh pathway activation in the anterior compartment of the wing discs [35], which induced overgrowth of the structure between Vein 2 and Vein 3 (Figure 6M, compare to a wild-type wing in 6L) or the thickness of Vein 3 (Figure 6N). Here, we found that overexpression of USP8 caused similar phenotypes (Figure 4P–Q). RNAi-mediated knockdown of USP8 severely disrupted the wing morphology (Figure 6O) and USP8C>S expression caused a more severe malformation of the wing as well as lethality of the pupa (unpublished data). The wing phenotypes correlated with the levels of Smo accumulation in wing discs where USP8RNAi, USP8C>S, or USP8 was expressed (Figure 6B, C, and F), suggesting that the adult wing phenotypes were caused, at least in part, by USP8-mediated regulation of Smo. Overexpression of USP8 elevated the level of Smo but did not induce ectopic Hh signaling activity in A compartment cells located away from the A/P boundary. This could be due to the inhibition of Smo by Ptc, since it has been shown that *ptc* mutant clones situated in the A-compartment of wing discs accumulate high levels of Smo [16]. To test this possibility, we expressed Flag-USP8 in the *ptc* mutant background and found that USP8 induced severe wing growth in the *ptc* heterozygote (Figure 6S, compared to 6R) and caused sick wings in the *ptc* homozygous background (Figure 6T). These results suggest that the USP8-mediated elevation of Smo is still inhibited by Ptc.

### USP8 Promotes Smo Signaling Activity

**Figure 7:**
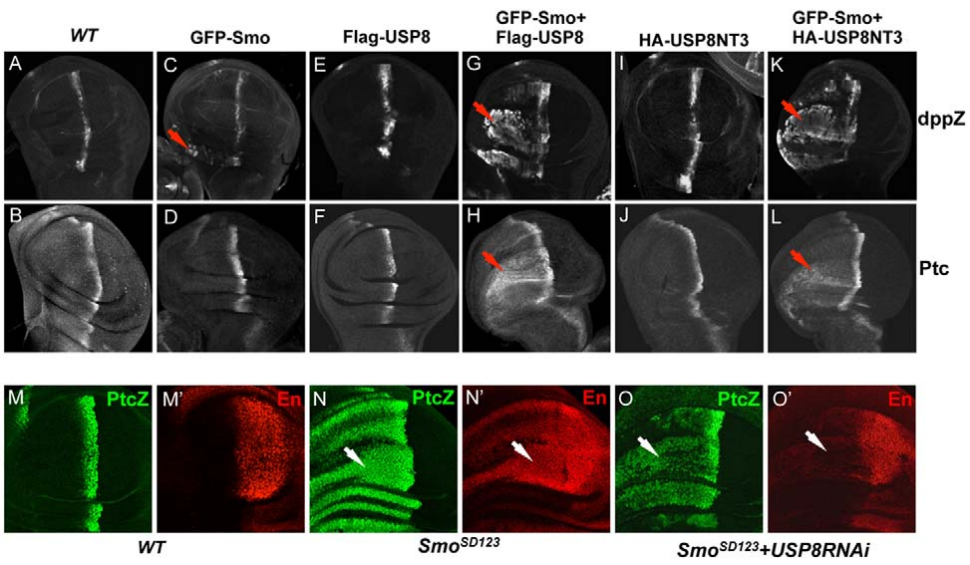
USP8 promotes Smo signaling activity. (A–B) Wild-type wing discs were stained with anti-β-Gal and anti-Ptc antibodies to show *dpp-lacZ* and endogenous Ptc expression, respectively. (C–L) Wing discs expressing GFP-Smo alone (C–D), Flag-USP8 alone (E–F), GFP-Smo and Flag-USP8 together (G–H), HA-USP8NT3 alone (I–J), or GFP-Smo and HA-USP8NT3 together (K–L) by MS1096 Gal4 were stained for dpp-LacZ or Ptc. The red arrows indicate the ectopic expression of either dpp-lacZ or Ptc. (M–M′) A wild-type wing disc was immunostained for ptc-lacZ and En. (N–O′) Wing discs expressing Smo^SD123^ alone or together with USP8RNAi by *MS1096* Gal4 was immunostained for ptc-lacZ and En. Knockdown of USP8 by RNAi attenuated the ectopic *ptc*-lacZ expression (arrow in O) and greatly reduced the ectopic *en* expression (arrow in O′).

Overexpression of USP8 caused an elevation of Smo in both A- and P-compartment cells but did not ectopically activate Hh target genes, such as *ptc*, in A-compartment cells located away from the A/P boundary (Figure 6G). This could have been due to insufficient levels of Smo. To test this possibility, we coexpressed Flag-USP8 with GFP-Smo, a construct that had been developed in an earlier study [35]. Co-expression of Flag-USP8 with GFP-Smo by *MS1096* Gal4 caused induction of *dpp-lacZ* and *ptc* expression in A-compartment cells both close to and away from the A/P boundary (Figure 7G–H), whereas the expression of Flag-USP8 alone did not induce ectopic *dpp-lacZ* and *ptc* expression (Figure 7E–F). Moreover, the expression of GFP-Smo only induced a low level of ectopic *dpp-lacZ* (Figure 7C) and no ectopic *ptc* expression (Figure 7D) in cells located away from the A/P boundary. Similar results were obtained when *act>CD2*>Gal4 was used to co-express Flag-USP8 with GFP-Smo, which suggested a cell-autonomous regulation (Figure S1A–B′). We also found that USP8 did not change the dominant negative activity of Smo^PKA123^ in wing discs (unpublished data), suggesting that phosphorylation and dimerization is required for Smo activation even though Smo can be stabilized by deubiquitination.

We had previously shown that Smo^SD123^ has constitutive cell-surface expression and signaling activity and is still regulated by Hh [12]. To test whether USP8 regulated the activity of Smo^SD123^ in vivo, Smo^SD123^ was expressed either alone or together with USP8RNAi and the expression of *ptc-lacZ* and *en* were examined. As shown in Figure 7N–O′, Smo^SD123^ showed constitutive signaling activity and induced the ectopic expression of *ptc-lacZ* and *en*. Knockdown of USP8 by RNAi attenuated the ectopic *ptc-lacZ* expression (Figure 7O) and greatly reduced the ectopic expression of *en* (Figure 7O′), which was likely due to the effect of USP8 regulating Smo^SD123^ ubiquitination (Figure 5B). Similar results were achieved by using *act>CD2*>Gal4 (Figure S1C–D′). These data suggest that Smo^SD123^ underwent a low level of ubiquitination, which prevents further activation.

### The Function of USP8 Domains in Regulating Smo

**Figure 8:**
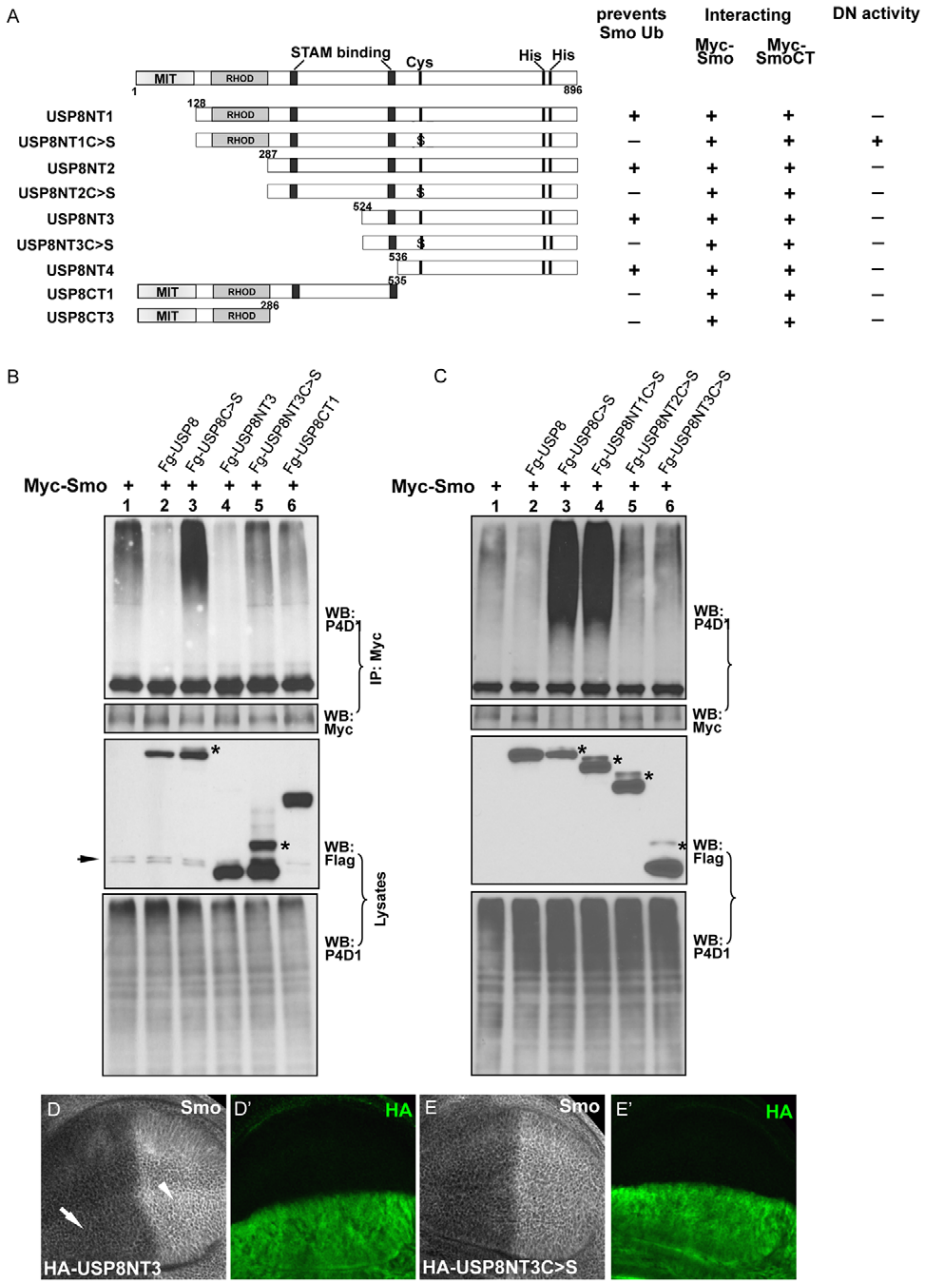
The deubiquitinase activity of USP8 variants. (A) A diagram of the USP8 protein and deletion constructs. The interaction between Smo and USP8 was determined by immunoprecipitation experiments shown in Figure S2. The activity of each USP8 construct was assessed in S2 cells by their ability to regulate the levels of Smo ubiquitination (shown below). MIT, microtubule interacting and transport domain; RHOD, Rhodanese homology domain. (B) S2 cells were transfected with Myc-Smo and the indicated USP8 constructs followed by the immunoprecipitation assay to detect Smo ubiquitination. The expression of USP8 variants was detected by a Western blot of lysates with an anti-Flag antibody (lower-middle panel). The arrow indicates a non-specific band and the asterisks indicate a possible modification of USP8C>S or USP8NT3C>S. USP8NT3C>S and USP8CT1 did not regulate Smo ubiquitination (lanes 5 and 6, top panel). (C) The effects of USP8 mutants on Smo ubiquitination were examined in S2 cells by the immunoprecipitation assay. The asterisks indicate a possible modification of USP8. Of note, both USP8C>S and USP8NT1C>S have dominant negative effects on Smo ubiquitination (lanes 3 and 4, top panel), whereas USP8NT2C>S and USP8NT3C>S do not (lanes 5 and 6, top panel). (D–E′) Wing discs expressing USP8NT3 or USP8NT3C>S by *ap*-Gal4 was immunostained for Smo and HA. The arrow and arrowhead in (D) indicate elevated Smo accumulation in A- and P-compartment cells. Notably, expression of USP8NT3C>S has no effect on Smo accumulation.

**Figure 9:**
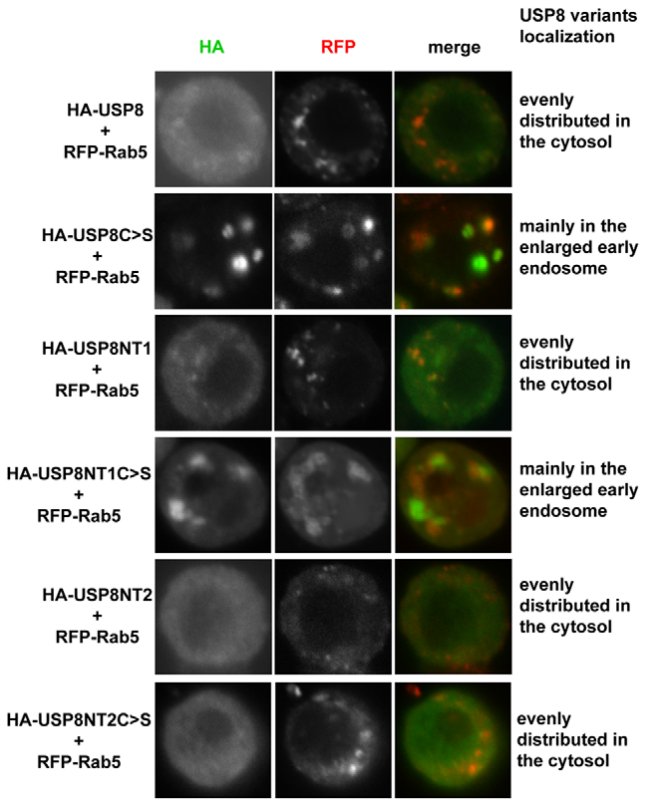
The localization of USP8 variants in S2 cells. S2 cells were cotransfected with USP8 constructs and RFP-Rab5 followed by immunostaining with an anti-HA antibody to label the expression of USP8 constructs. Rab5 marks the early endosome. Of note, the expression of USP8C>S and USP8NT1C>S causes enlarged early endosomes, and both forms of USP8 localize in these enlarged early endosomes. USP8, USP8NT1, USP8NT2, and USP8NT2C>S are evenly distributed in the cytosol without a specific pattern. USP8NT3 behaves like USP8NT2 and USP8NT3C>S behaves like USP8NT2C>S (unpublished data).

To further assess the function of USP8 in the regulation of Smo ubiquitination, we generated HA-tagged USP8 truncations and assessed their effects on Smo ubiquitination as well as their interaction with Smo in S2 cells (Figure 8A). The catalytic domain of USP8 (USP8NT3) was sufficient to reduce Smo ubiquitination (Figure 8B, lane 4, top panel), enhance the accumulation of endogenous Smo (Figure 8D), and increase the GFP-Smo-mediated induction of ectopic *dpp-lacZ* and *ptc* expression (Figures 7K–L, S1E–E′″). Similar to USP8, USP8NT3 alone did not induce any ectopic activation of Hh signaling in cells away from the A/P boundary (Figure 7I–J). We also examined the activity of USP8 mutants that contain the Cys572Ser mutations. Both USP8C>S and USP8NT1C>S had dominant negative effects, but USP8NT2C>S and USP8NT3C>S did not (Figure 8C). In addition, USP8NT3C>S had no effect on Smo accumulation in wing discs (Figure 8E–E′) or on GFP-Smo activity (unpublished data). These data suggest that the MIT and RHOD domains are required for USP8C>S to act in a dominant negative manner. We further examined the subcellular localization of USP8 variants and found that USP8C>S and USP8NT1C>S, but not the other forms, were primarily localized to Rab5-labeled and enlarged early endosomes, which was caused by the overexpression of these mutants (Figure 9). Rab5 is a small GTPase that is often used as a marker of the early endosome [36]. The results from the localization assay suggest that the N-terminal domains of USP8 are responsible for its accumulation in the early endosome and for the dominant negative activity.

We also carried out a series of immunoprecipitation experiments with S2 cells to map the USP8 domain that is responsible for the interaction with Smo. Collectively, the data showed that USP8NT3 was responsible for the interaction with Smo (Figures 8A and S2). Importantly, these results were in agreement with the finding that the catalytic domain of USP8 was capable of preventing Smo ubiquitination.

### USP8 Regulates Hh-Induced Cell Surface Accumulation of Smo

**Figure 10:**
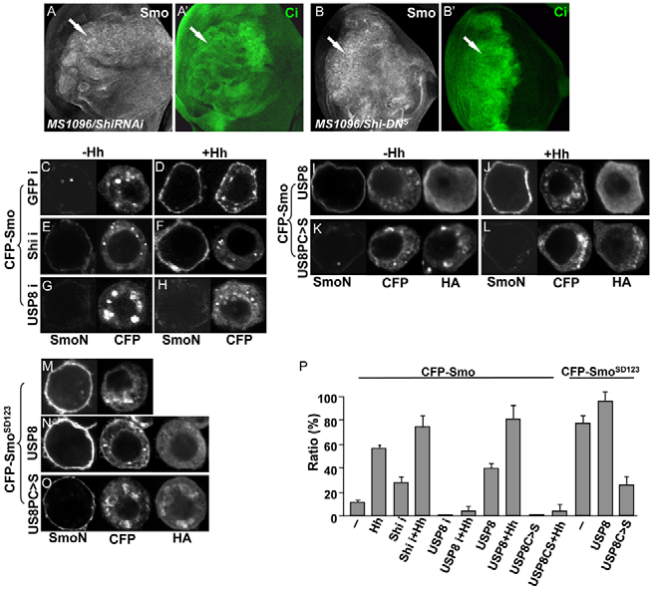
USP8 is required for Hh-induced Smo cell surface accumulation. (A–B′) Wing discs expressing *UAS-ShiRNAi* or *UAS- Shi-DN^S^* (a strong line) by *MS1096* Gal4 was stained for Smo and Ci. The arrows indicate the RNAi-mediated increase in accumulation of Smo and Ci. (C–O) CFP-Smo or CFP-Smo^SD123^ was transfected into S2 cells and the cells were treated with GFP dsRNA, Shi dsRNA, USP8 dsRNA, or Hh-conditioned medium. Smo that localized to the cell surface was visualized by immunostaining with an anti-SmoN antibody before membrane permeabilization. The total amount of expressed Smo was indicated by the CFP signal. Representative images are shown here. (P) Quantification analysis of the percentage of Smo on the cell surface (mean ± s.d.; *n*≥15). Ratio (%) = (cell surface signal/whole cell signal)×100.

In the absence of Hh, Smo remains in an unphosphorylated or hypophosphorylated state and can be removed from the cell surface by endocytosis. To confirm this presumption, we examined Smo levels in wing discs when the endocytosis machinery was inactivated. Shibire (Shi) is the *Drosophila* homolog of the dynamin GTPase that plays an essential role in regulating the internalization and sorting of membrane proteins [37] and is required for Ptc endocytosis [38]. We found that the accumulation of Smo was elevated in wing discs expressing Shi RNAi (Figure 10A) or expressing a dominant negative Shi (Shi-DN, Figure 10B), suggesting that Smo may function through Shi-mediated endocytosis. In addition, we found that the inactivation of Shi caused an increase in Ci (Figure 10A′ and B′), which suggested that Shi has a negative role in Hh signaling.

We next wished to assess whether the accumulation of Smo induced by the overexpression of USP8 or the inactivation of Shi was due to changes in the cell surface accumulation of Smo. CFP-Smo was transfected into S2 cells that were treated with GFP dsRNA (control), USP8 dsRNA, or Shi dsRNA and then treated with Hh-conditioned medium or control medium. Consistent with our previous observations [31], Hh stimulation causes the accumulation of Smo on the cell surface in the presence of control dsRNA (Figure 10D). Interestingly, the cell surface accumulation of Smo was elevated by the RNAi-mediated reduction of Shi (Figure 10E–F) or by the overexpression of USP8 (Figure 10I–J) in both the presence and absence of Hh stimulation. In contrast, the cell surface accumulation of Smo was reduced by an RNAi-mediated reduction of USP8 (Figure 10H) or by the overexpression of USP8C>S (Figure 10L) even in the presence of Hh stimulation. Without Hh treatment, Smo cell surface accumulation was barely detectable (Figure 10C, G, and K). These data suggest that USP8 promotes the cell surface accumulation of Smo, and that Shi mediates Smo endocytosis.

We have previously shown that Smo^SD123^ has constitutive cell surface expression [12]. To examine if USP8 regulates the cell surface accumulation of Smo^SD123^, we co-transfected CFP-Smo^SD123^ with USP8 or USP8C>S in S2 cells. The cell surface accumulation of Smo^SD123^ increased in the presence of USP8 (Figure 10N) and was reduced in the presence of USP8C>S or USP8 RNAi (Figure 10O, unpublished data), which suggested that the decrease in Smo^SD123^ activity (Figure S1D–D′) was due to the attenuation of cell surface accumulation of Smo^SD123^. Consistent with these data, the overexpression of USP8 reduced Smo^SD123^ ubiquitination, whereas the inactivation of USP8 increased Smo ubiquitination (Figure 5B). The quantification analysis of Smo cell surface accumulation further supported our findings (Figure 10P).

### USP8 Prevents the Localization of Smo to the Early Endosome

**Figure 11:**
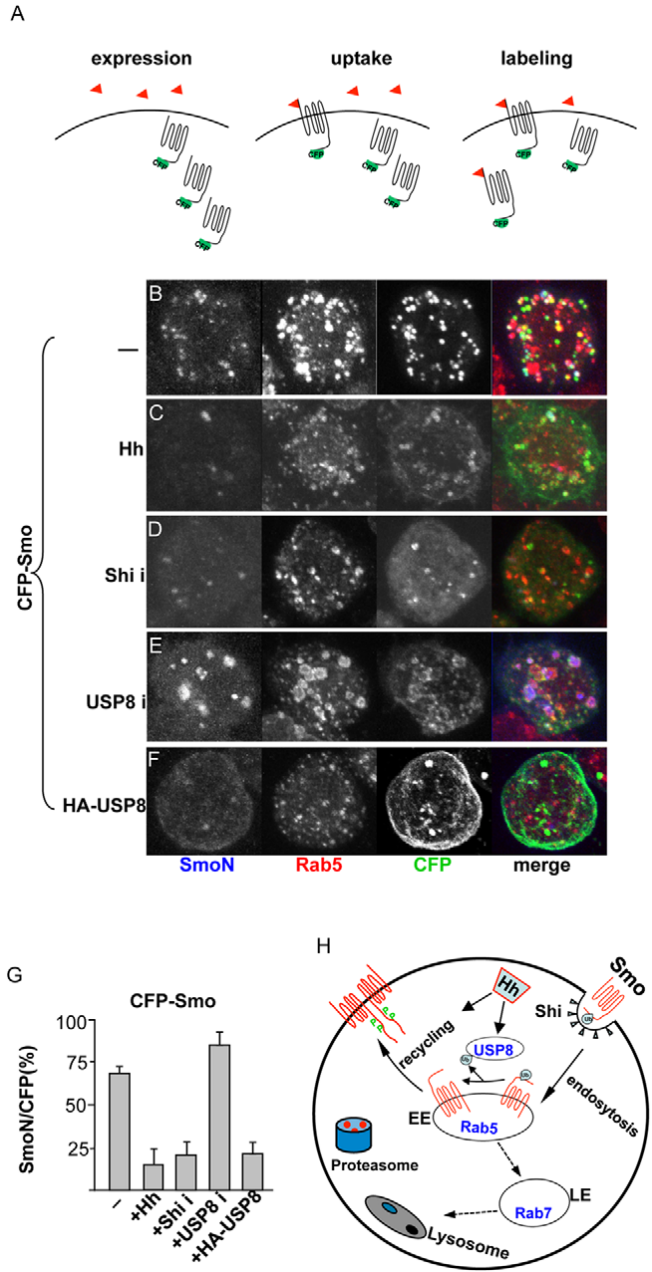
USP8 prevents Smo localization in the early endosome. (A) Illustration of the antibody uptake experiment. The red arrowheads indicate the route of the endocytosed anti-SmoN antibody. (B–F) S2 cells were transfected with CFP-Smo and treated with (B) control medium, (C) Hh-conditioned medium, (D) Shi dsRNA, (E) USP8 dsRNA, or (F) co-transfected with HA-USP8. The images shown here are from intensity projection over the *z*-axis. Hh largely inhibited the endocytosis of Smo, as indicated by the co-localization of Rab5 and SmoN (C). Reduction of Shi by RNAi and overexpression of USP8 have similar effects in the prevention of Smo localization in the early endosomes that are labeled by Rab5 staining (D and F). Knockdown of USP8 by RNAi caused the accumulation of Smo in enlarged early endosomes (E). Rab5 positive endosomes were identified based on Z-series confocal images of S2 cells stained with anti-Rab5 antibody. (G) The percentage of Smo that co-localized with Rab5 (SmoN staining) compared to the total amount of Smo (CFP signal) calculated from (B–F) (*n*≥10). (H) A model for Smo trafficking. USP8 is required for the Hh-induced cell surface accumulation of Smo. Hh blocks Shi-mediated Smo endocytosis. USP8 prevents Smo localization in the early endosome by deubiquitinating Smo, and promotes Smo phosphorylation and activation.

To determine whether the cell surface accumulation of Smo was due to a change in subcellular localization, we performed an antibody uptake assay with the anti-SmoN antibody to examine whether internalized Smo co-localized with Rab5. We hypothesized that blocking endocytosis may decrease Smo localization to the early endosomes, while inhibiting Smo recycling may lead to an accumulation of Smo in the early endosomes. As shown in Figure 11B, 99% of the internalized Smo protein co-localized with Rab5 positive particles, which occupied nearly 70% of the endosomes (Figure 11B). We also found that Hh treatment inhibited Smo endocytosis and reduced the ratio of Rab5-localized Smo (Figure 11C), which was consistent with our previous observation that Smo internalization is largely inhibited by Hh [12]. In addition, most Smo^SD123^ was not internalized (unpublished data). Therefore, Hh treatment and phosphorylation of Smo likely blocked Smo endocytosis. Moreover, Shi RNAi attenuated the localization of Smo in the early endosomes (Figure 11D), whereas USP8 RNAi elevated the localization (Figure 11E). The overexpression of USP8 decreased the amount of Smo that co-localized with Rab5 (Figure 11F), which suggested that USP8 may prevent the accumulation of Smo in early endosomes and therefore promote the cell surface accumulation of the protein (Figure 10I–J) as well as increase the signaling activity (Figure 7G–H). We also found that inactivation of USP8 by RNAi or by USP8C>S overexpression caused an enlargement of the early endosome (Figure 11E and Figure 9), which was consistent with a previous finding that USP8 deficient mouse primary cells exhibit enlarged early endosomes [29]. In addition, quantification of the amount of Smo that co-localized with Rab5 supported these conclusions (Figure 11G). Taken together, our data suggest that USP8 up-regulates the cell surface accumulation and signaling activity of Smo by deubiquitinating the protein (Figure 11H).

## Discussion

Smo is a key Hh signal transduction molecule located on the plasma membrane and there is a good correlation between the cell surface accumulation and signaling activity. However, little is known regarding the dynamic activation of Smo or the factors that regulate Smo trafficking. The present study has established that the multi-monoubiquitination of Smo is a reversible process that is impeded by an upstream signal. Moreover, this work has shown that a specific deubiquitinase inhibits Smo ubiquitination. Using an RNAi screen in both the *Drosophila* wing and cultured cells, we identified and characterized USP8 as the DUB that is required for Smo deubiquitination, accumulation, and activity.

### Smo Ubiquitination

In this study, we have uncovered that Smo is multi-monoubiquitinated and that the ubiquitination of Smo is reduced by Hh. However, it is currently unknown how Smo is ubiquitinated. Both *Drosophila* and vertebrate Smo contain multiple Lys residues that are highly conserved among various species [39], but it is not clear whether these residues can act as linkages for Ub. In an effort to characterize Smo ubiquitination, we mutated many of the conserved Lys residues in Smo C-tail and examined their ubiquitination by an immunoprecipitation assay and tested in vivo activities by expressing them at the equivalent levels in wing discs. However, we did not observe any changes in ubiquitination or activity (unpublished data). It is likely that monoubiquitination occurs at many sites on Smo, which requires the simultaneous mutation of most sites in order to detect an ubiquitination change.

In regards to the ubiquitination pattern of Smo, although multi-monoubiquitination is probably the predominant form, poly-ubiquitination may still occur. In fact, Li et al. (this issue) found that a small fraction of Smo was poly-ubiquitinated, which promoted Smo degradation through the proteasome, suggesting that Smo underwent both multi- and poly-ubiquitination. The specific factors, such as ubiquitin ligase(s), involved in Smo ubiquitination are also unknown. Identification of these pathway components may explain why Smo is either multi-monoubiquitinated or poly-ubiquitinated under different circumstances.

Although the loss-of-function of USP8 can block Hh-induced cell surface accumulation of Smo, we found that USP8 only stabilizes Smo but does not regulate Smo phosphorylation in the absence of Hh (Figure 5G), suggesting that the regulation of Smo by USP8 is downstream of Smo phosphorylation. Consistent with this idea, we found that the phospho-mimetic Smo^SD123^ was still regulated by USP8 (Figures 5B, 7O–O′, 10N–O, S1D–D′). USP8 likely plays an additional role in blocking Smo degradation. However, based on our current knowledge and available tools, we cannot clearly separate these roles of USP8 in regulating Smo.

It has been shown that the Smo C-tail mediates binding with Cos2, Fu, and PP4 [30],[35],[40]–[42]. In this study, we add USP8 as another protein that binds to the Smo C-tail region between amino acids 625–753, which includes the three PKA and CK1 phosphorylation clusters. It is likely that phosphorylation at this region promotes the binding of USP8. In support of this notion, we found that Hh promotes the interaction between Smo and USP8 (Figure 5D). We further found that a phospho-mimetic mutation of Smo promotes its binding with USP8 (Figure 5E–F). Previously, we created a deletion in Smo to avoid protein binding and found that it affected the in vivo activity of Smo [30]. One would expect that deletion of the USP8 binding domain may lead to hyper-ubiquitination and thus inactivation of Smo, because this binding domain contains the phosphorylation clusters that are essential for Smo activation. However, there may be ubiquitination sites within this region, and we have not as yet identified the ubiquitination residues in Smo.

### Smo Cell-Surface Accumulation and Subcellular Trafficking

Phosphorylation of Smo has been shown to be a crucial protein modification event for the activation of Smo in response to Hh. In this study, we found a role of ubiquitination in Smo trafficking inside the cell by demonstrating that the removal of Ub from Smo prevents localization of the protein to early endosomes, and the addition of an Ub signal promotes Smo localization in the late endosome. Thus, ubiquitination of Smo most likely promotes Smo endocytosis in a similar manner as that seen for other plasma membrane proteins where ubiquitination regulates endocytosis [24]. We have also discovered an additional molecular mechanism of Smo activation by demonstrating that Hh activates Smo by inhibiting its ubiquitination. We sought to understand the mechanism by which Hh reduces Smo ubiquitination and found that the inactivation of USP8 by RNAi or by the expression of a dominant negative USP8 abolished the effects of Hh on Smo ubiquitination. These data suggest that USP8 is required for the Hh effects on Smo ubiquitination. The findings that Hh promotes the interaction between Smo and its DUB, and that phosphorylation of Smo further promotes this interaction, helped us to better understand the underlying molecular mechanisms. In addition, Hh may regulate the assembly of the trafficking complexes, which could include USP8. One piece of evidence came from our finding that the deletion of the MIT and the RHOD domains in USP8C>S abolished the dominant negative effect, indicating that the N-terminal portion of USP8 (aa1–287) was necessary for the mutant to act as dominant negative protein, possibly through the interaction with the component(s) of the endosomal sorting complex required for transport (ESCRT). In support of this model, mutating *hrs* has been shown to cause an accumulation of Smo in *Drosophila* ovarian follicle cells [43] or in wing imaginal discs (Li et al., this issue; our unpublished observation), and therefore Hrs may recognize ubiquitinated Smo and target Smo to the lysosome for degradation. It is also possible that Hh may regulate Smo ubiquitin ligase(s) that are as yet undefined.

Here, we have shown that Smo accumulates on the cell surface when Shi is inactivated, suggesting that Smo functions through Shi-mediated endocytosis. Although our observations support the notion that USP8 deubiquitinates Smo and prevents localization to early endosomes, we are not suggesting that USP8 play an exclusive role in the inhibition of Smo endocytosis. It is also possible that USP8 promotes Smo recycling to the cell surface. Future studies will focus on determining the route of Smo movement to the cell surface.

### The Role of USP8 in Regulating Smo

USP family members have been shown to be involved in the regulation of different cellular pathways [44]. USP8 was first described as a growth-regulated ubiquitin isopeptidase that plays a possible role in the control of mammalian cell proliferation [45]. There is increasing evidence that USP8 has a role in endosomal sorting [28],[29],[46]. The identification and characterization of USP8 in this study as a DUB involved in the reduction of Smo ubiquitination has provided the opportunity to further understand the functions of USP8 in regulating membrane protein trafficking. We have characterized the role of USP8 in the regulation of Smo cell surface expression that is critical for Smo activation. We have also provided evidence that USP8 is required for Hh-induced Smo accumulation. However, although the overexpression of USP8 increased the levels of Smo in vivo, it failed to induce Hh target gene expression in A-compartment cells located away from the A/P boundary. Our explanation is that the increase in Smo levels that was induced by USP8 was still inhibited by Ptc, since we found that USP8 induced more severe overgrowth phenotypes in the *ptc* mutant background (Figure 6R–T). It is also possible that USP8 might have additional function(s) in the Hh pathway by targeting other component(s).

## Materials and Methods

### Constructs, Mutants, and Transgenes

The Myc-Smo, Myc-SmoCT, and Myc-SmoΔCT constructs have been previously described [35]. To construct Flag-tagged DUBs, we obtained full-length cDNA from either DGRC or by RT-PCR from fly embryonic RNA, amplified the cDNA fragments, and sub-cloned them into the 2xFlag-UAST vector. Flag-USP8C>S was generated by site-directed mutagenesis and sub-cloned into the 2xFlag-UAST. The HA-tagged USP8, USP8C>S, USP8NT1 and USP8NT1C>S (aa 128–896), USP8NT2 and USP8NT2C>S (aa 287–896), USP8NT3 and USP8NT3C>S (aa 523–896), USP8CT1 (aa 1–534), and USP8CT3 (aa 1–286) were constructed by inserting the coding sequences into the 2xHA-UAST vector. Myc-SmoUb and Myc-SmoCFP were constructed by sub-cloning the Ub and CFP sequences in frame with the C-terminus of Myc-Smo, respectively. To generate GFP-SmoUb, the GFP sequence was used to replace Myc in Myc-SmoUb. Smo variants with substitutions at Lysine residues were generated by site-directed mutagenesis in the background of Myc-Smo. To construct Myc-hSmo, 6xMyc tag was introduced into the *Xho*I site after the signal peptide of human Smo, and then subcloned into pCDNA. RFP-Rab5 and RFP-Rab7 were constructed by fusing the RFP sequence in frame to the N-terminus of Rab5 or Rab7. The HA-tagged Ub and Ub mutants were constructed by swapping the cDNA fragments into the 2xHA-UAST vector. UbK0 bears K to R mutations at all of the lysine residues in Ub. The UbK48 and UbK63 constructs have mutations at all of the lysine residues, with the exception of K48 and K63, respectively. The UbK11R, UbK29R, UbK48R, and UbK63R have single K to R mutations as indicated. The generation of transgenes at the 75B1 attP locus (resulting VK5 lines) has been previously described [30]. The VK5-Flag-USP8, USP8C>S, HA-USP8NT3, USP8NT3C>S, Myc-SmoUb, and Myc-SmoCFP transgenes were generated using the same approach. The RNAi lines that targeted each DUB in the *Drosophila* genome were obtained from the Vienna *Drosophila* RNAi Center (VDRC) (Table S1) [47]. USP8 RNAi lines (v8931 and v107623) were consistent in terms of the adult wing phenotypes and the effects on Smo accumulation in wing discs. Shi RNAi lines (v3798 and v3799) obtained from the VDRC gave rise to similar phenotypes when driven by *MS1096* Gal4. *UAS-Shi-DN*, *UAS-Rab5*, *ptc^H84^*, and *ptc^IN108^* strains have been previously described (Flybase) [36],[48],[49]. The *usp8* mutant (*usp8KO*) and *pka* mutant (*DC0^E95^*) have been previously described [12],[28]. To generate mutant clones, FRT/FLP-mediated mitotic recombination was used for the following genotypes: *usp8* clone: *yw hsp-flp/+* or *Y*; *FRT82 usp8Δ/FRT82 hsp-GFP*, or *yw hsp-flp/+* or *Y*; *FRT82 usp8-KO/FRT82 ubi-lacZ*. *DC0* clone: *yw hsp-flp/+* or *Y*; *DC0^E95^ stc FRT40/hsp-GFP FRT40*. The *MS1096* Gal4, *act>CD2*>Gal4, *UAS-GFP-Smo*, *dpp-lacZ*, and *ptc-lacZ* fly lines have been previously described [35].

### Antibody Production

The USP8 antibody was generated by Genemed Synthesis Inc. in rabbits immunized with peptides corresponding to aa1–16 and 452–470 of USP8. The anti-SmoP antibody was generated by the same company to specifically detect Smo phosphorylation at the 2^nd^ cluster (anti-SmoP). Antigen peptide CRHVSVESRRN(pS)VD(pS)QV(pS)VK was injected into the rabbits, the serum was affinity-purified with the antigen, and the flow-through was kept as a control antibody against non-phosphorylated peptide (Fan et al., unpublished).

### Cell Culture, Transfection, Immunoprecipitation, Western Blot, and GST Fusion Protein Pull-Down

S2 cells were cultured as previously described [31]. Transfections were carried out using Effectene transfection reagent (Qiagen). The immunoprecipitation and immunoblot analysis were performed using a standard protocol. Faf, USP8, CG12082, USP7, CG7288, CG9448, NOT, CG4165, Ec, CYLD, and USP36 dsRNA were synthesized against nucleotides 2310–2910, 821–1670, 151–750, 181–750, 151–750, 175–670, 481–1070, 151–730, 2942–3542, 21–596, and 932–1532 of each corresponding gene, respectively. GFP dsRNA synthesis and the method for treating S2 cells with dsRNA have been previously described [31]. Hh treatment was carried out by using 60% Hh-conditioned medium unless otherwise specified. NH_4_Cl (Sigma-Aldrich) was used as a lysosomal inhibitor at a final concentration of 10–15 mM for 24 h before harvesting the cells. H-89 and CK1-7 were used as the PKA and CK1 inhibitors at a concentration of 10 µM and 100 µM, respectively, as previously described [12]. NIH3T3 cells were cultured in DMEM medium containing 10% bovine calf serum in a humidified incubator with 5% CO2. After 24 h post-transfection, the medium was changed to DMEM with 0.5% bovine calf serum with or without recombinant mouse Shh (R&D Systems). HEK293T cells were cultured in DMEM containing 10% fetal bovine serum (FBS). Transfection of NIH3T3 and HEK293T cells were carried out using FuGENE6 (Roche). GST-Smo was constructed by fusing Smo aa625–753 to the GST backbone. The GST fusion protein pull-down has been previously described [30].

### In Vivo Ubiquitination Assay

To examine the levels of ubiquitinated Smo, S2 cells were transfected with Myc-Smo and then lysed with denaturing buffer (1% SDS, 50 mM Tris, pH 7.5, 0.5 mM EDTA, and 1 mM DTT) and incubated at 100°C for 5 min. The lysates were then diluted 10-fold with regular lysis buffer containing 1.5 mM MgCl_2_ and subjected to immunoprecipitation with an anti-Myc antibody. The protein was then resolved on an 8% SDS-PAGE, and an immunoblot was performed using an anti-ubiquitin antibody (to detect the endogenous Ub) or an anti-HA antibody (to detect the HA-Ub or HA-Ub mutants). The ubiquitination of Myc-hSmo from NIH3T3 cells was examined by the same immunoprecipitation assay as used for Myc-Smo. The antibodies used for Western blot included the following: mouse anti-Myc (9E10, Santa Cruz, 1∶5,000), anti-Flag (M2, Sigma, 1∶10,000), anti-β-tubulin (DSHB, 1∶2,000), anti-ubiquitin (P4D1, Santa Cruz, 1∶500), anti-GFP (Millipore, 1∶1,000), and anti-GST (Santa Cruz, 1∶10,000); rabbit anti-USP8 (this study, 1∶200), and anti-SmoP (Fan et al., unpublished, 1∶50).

### Cell Surface Staining, Antibody Uptake, and Protein Localization Assays

The cell-based assay to detect Smo cell surface accumulation was carried out by immunostaining with an anti-SmoN antibody (DSHB, 1∶100) before cell permeabilization [31]. For the antibody uptake assay, transfected S2 cells were incubated with the anti-SmoN antibody (DSHB, 1∶100, concentrated) in S2 cell medium for 2 h at 25°C, fixed, and permeabilized. The fixed cells were then stained with an anti-Rab5 antibody (Abcam, 1∶300), washed, and then stained with a secondary antibody. Z-scan sections of regular cells (12–14 µm in diameter) were taken at 0.7 µm by confocal imaging (Olympus Fluoview, Ver.1.7c) to monitor the Rab5-positive endosomes and to quantify the Smo that was endocytosed after intensity projection over the *z*-axis. CFP-Smo signals were used to normalize the overall levels of transfected Smo. For quantification, an autoradiography densitometric analysis was performed using Metamorph software. Protein co-localization assays were carried out by co-transfecting S2 cells with Smo or USP8 constructs in combination with RFP-Rab7 or RFP-Rab5, followed by immunostaining and confocal analysis. Where appropriate, the experimental groups were compared using the Student's two-tailed *t* test, with significance defined as *p*<0.05.

### FISH, Embryo Staining, and Wing Disc Immunostaining

For fluorescence in situ hybridization (FISH), *yw* wing discs from third instar larvae were collected and USP8 RNA probes were prepared according to the instruction of the DIG RNA labeling kits (Roach). After fixation, hybridization, and post-hybridization washes, wing discs were subjected to secondary antibody incubation and then washed with PBT buffer. For embryo immunostaining, stage 11 embryos from specific genotypes were dechorionated, fixed in solution containing 80% Heptane, and immunostained with the indicated antibodies. *Act5C*-Gal4 (Flybase) was used to drive the expression of UAS-USP8RNAi or UAS-USP8C>S in embryos. A standard protocol was used for the immunostaining of wing discs from late third instar larvae. Antibodies used in this study were as follows: mouse anti-Myc (9E10, Santa Cruz, 1∶50), anti-Flag (M2, Sigma, 1∶150), anti-ubiquitin (FK2, BIOMOL, 1∶50), anti-ubiquitin (P4D1, Santa Cruz, 1∶50), anti-SmoN (DSHB, 1∶10), anti-Ptc (DSHB, 1∶10), and anti-En (DSHB, 1∶20); rabbit anti-Flag (ABR, 1∶150), anti-HA (Y-11, Santa Cruz, 1∶50), anti-β-Gal (Cappel, 1∶1,500), and anti-GFP (Clontech, 1∶500); and rat anti-Ci, (2A, DSHB, 1∶10).

## Supporting Information

Figure S1 Mutating PKA in wing disc accumulates ubiquitinated protein. (A–A″) A wing disc carrying *DC0* clones was stained with anti-Smo (red) and anti-GFP (green) antibodies. *DC0* clones lack GFP expression. The arrow in (A) shows the reduced Smo accumulation in P-compartment cells. (B–C″) Wing discs carrying *DC0* clones were stained with anti-Ub FK2 or P4D1 antibody. The anti-Ub FK2 antibody labeled the ubiquitinated proteins and the anti-Ub P4D1 labeled the total levels of Ub in wing discs. Ub-FK2 staining was elevated in cells mutating *DC0* (arrow in B).
(TIF)

Figure S2 USP8 promotes Smo signaling activity (related to Figure 7). (A–B′) Wing discs expressing GFP-Smo alone or in combination with Flag-USP8 expressed by *act>CD2*>Gal4 were stained for ptc-lacZ. Clones are marked by GFP expression. The arrowheads in (A) and (B) indicate ectopic ptc-lacZ expression in A-compartment cells located near the A/P boundary. The arrow in (B) indicates the ectopic ptc-lacZ expression in cells located away from the A/P boundary. (C–D′) Wing discs expressing Smo^SD123^ alone or in combination with USP8RNAi expressed by *act>CD2*>Gal4 were stained for ptc-lacZ and En. The arrows in (C) and (C′) indicate the ectopic activation of Ptc and En by Smo^SD123^. The arrows in (D) and (D′) indicate the reduction of ptc-lacZ and En expression by USP8RNAi. (E–E′″) A wing disc expressing GFP-Smo together with HA-USP8NT3 by *act>CD2*>Gal4 was stained for HA and Ptc-lacZ. The clones are marked by GFP expression. The arrow indicates the ectopic Ptc-lacZ expression that was induced by the co-expression of GFP-Smo with USP8NT3.
(TIF)

Figure S3 The interaction between Smo and USP8 (related to Figure 8). (A) S2 cells were co-transfected with Myc-Smo and the indicated USP8 constructs, followed by immunoprecipitation with the anti-Myc antibody and Western blot with either the anti-HA antibody to detect the bound USP8 (top panel) or with the anti-Myc antibody to detect the expression of Myc-Smo (middle panel). IgG served as the loading control. Cell extracts were also subjected to a direct Western blot with the anti-HA antibody to examine the expression of HA-tagged USP8 or the truncated forms (bottom panel). USP8, USP8NT1, NT2, NT3, and NT4 were pulled down by Myc-Smo but not USP8CT1 or USP8CT3. See Figure 4E for the diagram of the USP8 constructs. (B) S2 cells were transfected with the indicated constructs followed by immunoprecipitation and Western blot with the indicated antibodies. The arrow in the top panel indicates full-length USP8, and the arrowhead indicates USP8NT3, which were pulled down by Myc-Smo or Myc-SmoCT (lanes 1, 3, 4, and 6) but not by Myc-SmoΔC (lanes 2 and 5). The middle panel indicates the expression and input of Smo protein. The bottom panel indicates the expression of USP8 that was detected by Western blot with the anti-HA antibody. (C) S2 cells were co-transfected with Myc-SmoCT and the USP8 variants, followed by immunoprecipitation with the anti-Myc antibody and Western blot with either the anti-HA antibody to detect SmoCT-bound USP8 (top panel) or with the anti-Myc antibody to detect the expression of Myc-Smo (middle panel). IgG served as the loading control. Cell extracts were subjected to Western blot with the anti-HA antibody to examine the expression of HA-tagged USP8 or the truncated forms (bottom panel). USP8, USP8NT1, NT2, NT3, and NT4 were pulled down by Myc-SmoCT, but USP8CT1 and USP8CT3 were not. This result was similar to the interaction pattern of full-length Smo.
(TIF)

Table S1 In vivo RNAi-mediated reduction of DUBs in the *Drosophila* genome was performed by using the VDRC RNAi library. The asterisks indicate that the RNAi-mediated reduction of these DUBs via *MS1096* Gal4 induced adult wing phenotypes.
(XLS)

## Abbreviations

Ci: cubitus interuptus
CK1: casein kinase 1
Cos2: costal 2
Dpp: decapentaplegic
DUB: de-ubiquitinating enzyme
Gprk2: G protein-coupled receptor kinase 2
HA: hemagglutinin
Hh: Hedgehog
NH4Cl: ammonium chloride
PKA: protein kinase A
Ptc: Patched
RTKs: receptor tyrosine kinases
Shi: shibire
Smo: Smoothened
Ub: ubiquitin
USP8: ubiquitin-specific protease 8
