# Supplementary figures and images for: USP8 Promotes Smoothened Signaling by Preventing Its Ubiquitination and Changing Its Subcellular Localization
Source: PLoS Biol. 2012 Jan 10;10(1):e1001238. doi: 10.1371/journal.pbio.1001238 (PMC3254663; doi:10.1371/journal.pbio.1001238)

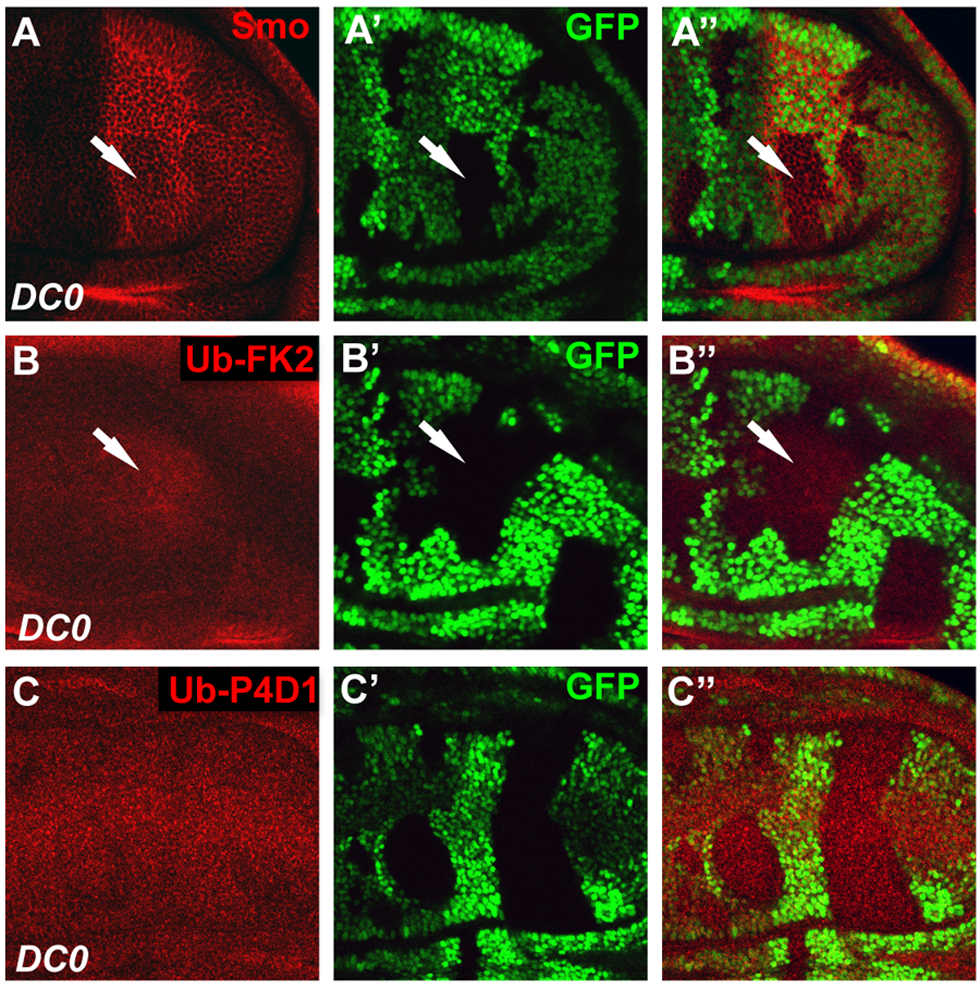

Supplement: Figure S1 — Mutating PKA in wing disc accumulates ubiquitinated protein. (A–A″) A wing disc carrying DC0 clones was stained with anti-Smo (red) and anti-GFP (green) antibodies. DC0 clones lack GFP expression. The arrow in (A) shows the reduced Smo accumulation in P-compartment cells. (B–C″) Wing discs carrying DC0 clones were stained with anti-Ub FK2 or P4D1 antibody. The anti-Ub FK2 antibody labeled the ubiquitinated proteins and the anti-Ub P4D1 labeled the total levels of Ub in wing discs. Ub-FK2 staining was elevated in cells mutating DC0 (arrow in B). (TIF) [file pbio.1001238.s001.tif]

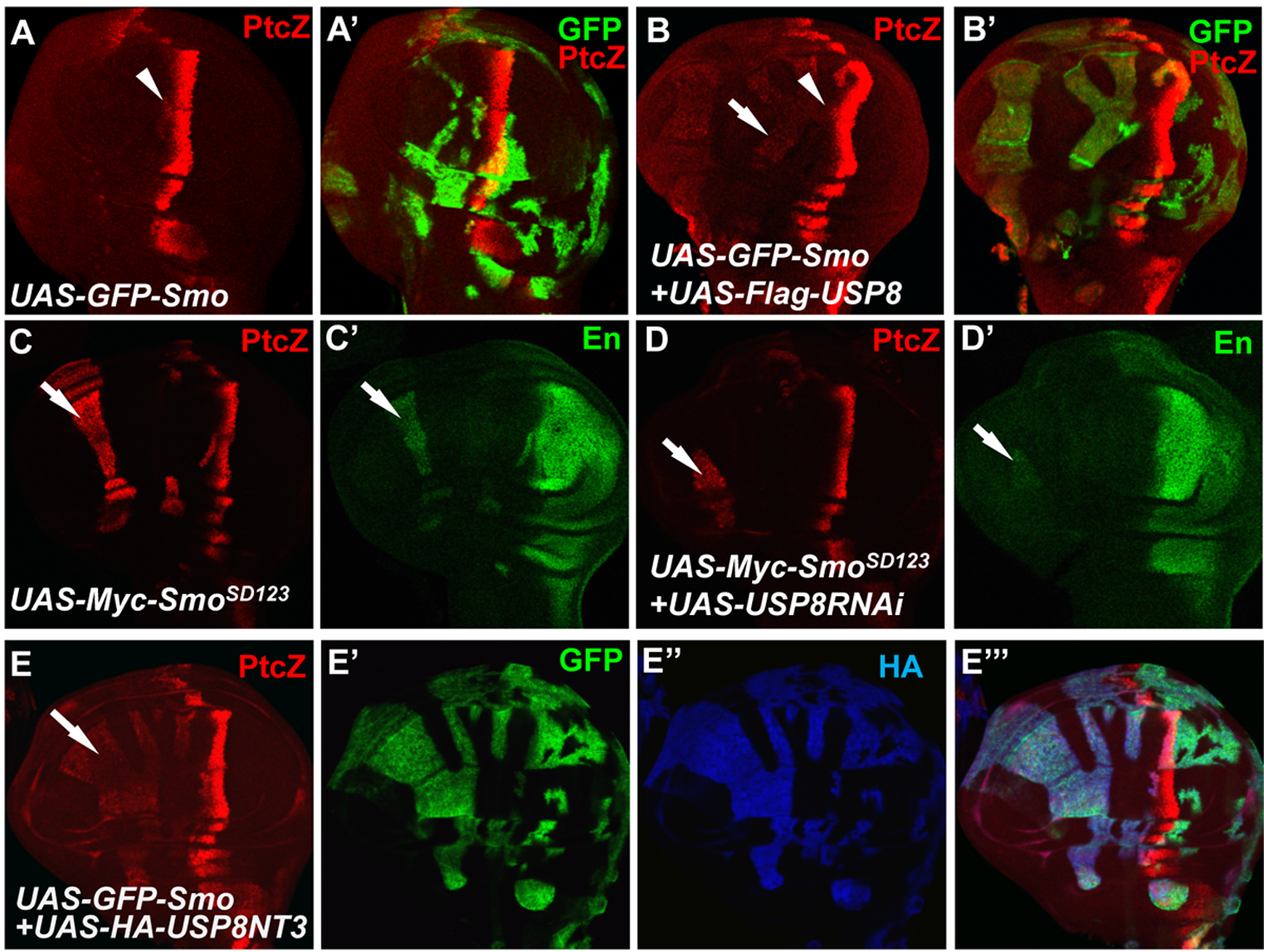

Supplement: Figure S2 — USP8 promotes Smo signaling activity (related to Figure 7). (A–B′) Wing discs expressing GFP-Smo alone or in combination with Flag-USP8 expressed by act>CD2>Gal4 were stained for ptc-lacZ. Clones are marked by GFP expression. The arrowheads in (A) and (B) indicate ectopic ptc-lacZ expression in A-compartment cells located near the A/P boundary. The arrow in (B) indicates the ectopic ptc-lacZ expression in cells located away from the A/P boundary. (C–D′) Wing discs expressing SmoSD123 alone or in combination with USP8RNAi expressed by act>CD2>Gal4 were stained for ptc-lacZ and En. The arrows in (C) and (C′) indicate the ectopic activation of Ptc and En by SmoSD123. The arrows in (D) and (D′) indicate the reduction of ptc-lacZ and En expression by USP8RNAi. (E–E′″) A wing disc expressing GFP-Smo together with HA-USP8NT3 by act>CD2>Gal4 was stained for HA and Ptc-lacZ. The clones are marked by GFP expression. The arrow indicates the ectopic Ptc-lacZ expression that was induced by the co-expression of GFP-Smo with USP8NT3. (TIF) [file pbio.1001238.s002.tif]

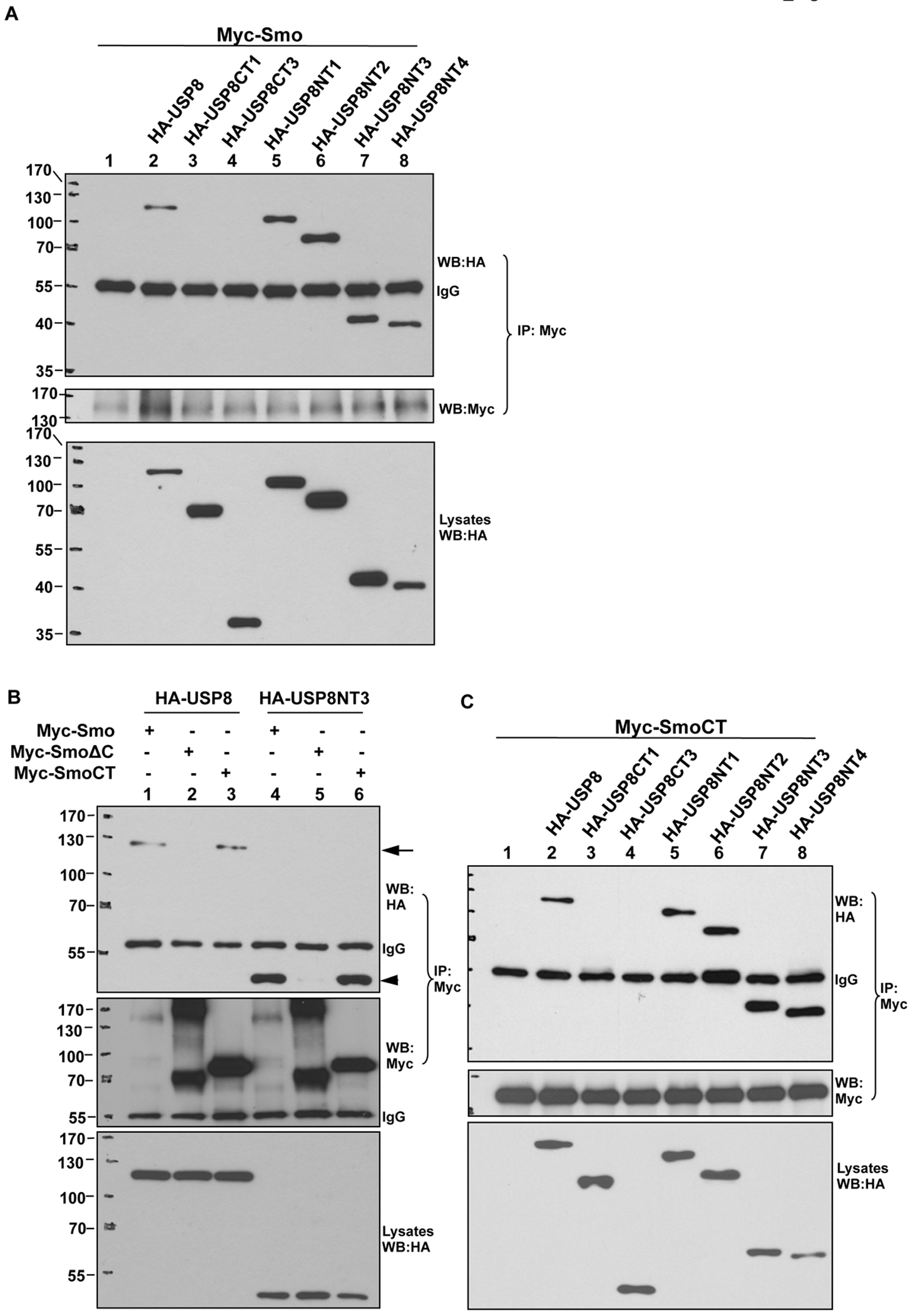

Supplement: Figure S3 — The interaction between Smo and USP8 (related to Figure 8). (A) S2 cells were co-transfected with Myc-Smo and the indicated USP8 constructs, followed by immunoprecipitation with the anti-Myc antibody and Western blot with either the anti-HA antibody to detect the bound USP8 (top panel) or with the anti-Myc antibody to detect the expression of Myc-Smo (middle panel). IgG served as the loading control. Cell extracts were also subjected to a direct Western blot with the anti-HA antibody to examine the expression of HA-tagged USP8 or the truncated forms (bottom panel). USP8, USP8NT1, NT2, NT3, and NT4 were pulled down by Myc-Smo but not USP8CT1 or USP8CT3. See Figure 4E for the diagram of the USP8 constructs. (B) S2 cells were transfected with the indicated constructs followed by immunoprecipitation and Western blot with the indicated antibodies. The arrow in the top panel indicates full-length USP8, and the arrowhead indicates USP8NT3, which were pulled down by Myc-Smo or Myc-SmoCT (lanes 1, 3, 4, and 6) but not by Myc-SmoΔC (lanes 2 and 5). The middle panel indicates the expression and input of Smo protein. The bottom panel indicates the expression of USP8 that was detected by Western blot with the anti-HA antibody. (C) S2 cells were co-transfected with Myc-SmoCT and the USP8 variants, followed by immunoprecipitation with the anti-Myc antibody and Western blot with either the anti-HA antibody to detect SmoCT-bound USP8 (top panel) or with the anti-Myc antibody to detect the expression of Myc-Smo (middle panel). IgG served as the loading control. Cell extracts were subjected to Western blot with the anti-HA antibody to examine the expression of HA-tagged USP8 or the truncated forms (bottom panel). USP8, USP8NT1, NT2, NT3, and NT4 were pulled down by Myc-SmoCT, but USP8CT1 and USP8CT3 were not. This result was similar to the interaction pattern of full-length Smo. (TIF) [file pbio.1001238.s003.tif]
